# What students want? Experiences, challenges, and engagement during Emergency Remote Learning amidst COVID-19 crisis

**DOI:** 10.1007/s10639-021-10747-1

**Published:** 2021-10-20

**Authors:** Rucha Tulaskar, Markku Turunen

**Affiliations:** Faculty of Information Technology and Communication Sciences, Tampere University, Tampere, Finland

**Keywords:** Emergency Remote Learning, COVID-19, Remote learning, Engagement, Challenges, Finland, India, Higher education, Tertiary education, Information Technology, Pandemic, Pedagogy, Learning experience

## Abstract

COVID-19 pandemic has affected the entire world in many ways. It has sparked a prominent pedagogical shift for university level students, as it has changed the way students learn, attend classes, or communicate with teachers. Globally, every student is forced to adopt Emergency Remote Learning (ERL) as a result of immediate transformation of physical classes into remote education. This two-fold study investigated the differences between traditional distance, online, and virtual learning solutions and the new Emergency Remote Learning (ERL) method for the university level education. Furthermore, a pragmatic mix-method study is conducted in the form of surveys, semi-structured interviews, and diary study spanning across 10 months of pandemic, to examine self-reported insights on ERL challenges, experiences, and learning engagement of the students from Finland and India. Cumulative findings suggest that scheduling, distractions, pessimistic emotions, longer durations, and concentration were the highest challenges faced by the students which impacted their learning experiences and engagement. The study also found that the ERL specific factors like low-interactivity, technical limitations, non-structured, and non-standardized methods had a prominent impact on the effectiveness of remote education. Furthermore, the study has suggested guidelines for improving remote learning experience as a futuristic solution beyond COVID-19 pandemic.

**Supplementary Information:**

The online version contains supplementary material available at 10.1007/s10639-021-10747-1.

## Introduction

In 2020, the world faced COVID-19 pandemic lockdown and by the end of March 2020, almost all the institutions were closed (UNESCO, 2020), affecting 70% of the student population around the world. This resulted in the highest ‘online movement’ in the history of education (Aristovnik et al., 2020). With minimal time to convert into remote learning, lack of planning and unfamiliarity of new online resources by students and teachers, this transition was different from conventional well-planned online learning experiences. This makeshift online learning is coined as Emergency Remote Learning (ERL) since it was delivered in response to a crisis or catastrophe (Hodges et al., 2020; Rahiem, 2020; Schultz, 2020; Vollbrecht, 2020).

Many researchers from around the world have already conducted studies on higher education during COVID-19 crisis. But the studies with focus on ‘pedagogy-in-pandemic’ are either [1] country specific—Indonesia (Rahiem, 2020), China (Cao et al., 2020; Xiao & Li, 2020), Philippines (Toquero, 2020), India (Kapasia et al., 2020) or [2] subject specific—chemistry (Riley & McNeil, 2020), Medical (Sahi et al., 2020) or [3] based on students’ life issues, wellbeing and engagement (Edelhauser E., 2020; Sahu P., 2020). Some studies have been done on the ‘emergency pedagogical shift’ (Toquero, 2020) and a every few studies focus on multi-country or multi-discipline contexts (Aristovnik et al., 2020; Reznik et al., 2020). Furthermore, almost all of them established their findings on quantitative data analysis. There are hardly any studies (Rahiem, 2020), which investigated more authentic insights gathered through qualitative methods in this one-of-a-kind situation. In addition, very few researchers have focused on comparing conventional remote learning methods with ERL which is an essential step to recommend solutions for improving ERL as a remote educational solution. Most importantly, a longitudinal study encompassing a longer period of time during the pandemic is certainly lacking, as most of the studies are conducted at the beginning of the pandemic resulting in small sample size and limited perspective towards pandemic pedagogy.

Current study aims at bridging above gaps by understanding the use of ERL during the COVID-19 crisis in tertiary education from various disciplines and two different geographical locations, investigating insights of students from a developed European country like Finland in contrast with a developing Asian country like India. The study was conducted in two parts. The first part investigates the similarities and the differences between conventional remote, online, and e-learning methods with ERL. The second part of the study applies a pragmatic mix-method encompassing surveys, semi-structured interviews, and diary study to investigate university students' self-reported perspectives on challenges, benefits, experiences, needs, and engagement while using ERL. Furthermore, a longitudinal approach of this study, conducted during the 10 months of pandemic, also compares the effects of long-term use of ERL and country specific findings. This study presents a novel and original contribution of students’ learning experiences, as the primary objective of this study and provides immediate access to the actual insights to help ensure efficacy of ongoing ERL in higher education and incorporate better programs for future ready versions. Finally, the study has suggested guidelines based on the findings to improve ERL as a potential remote learning solution in the near future.

## Background

**Fig. 1:**
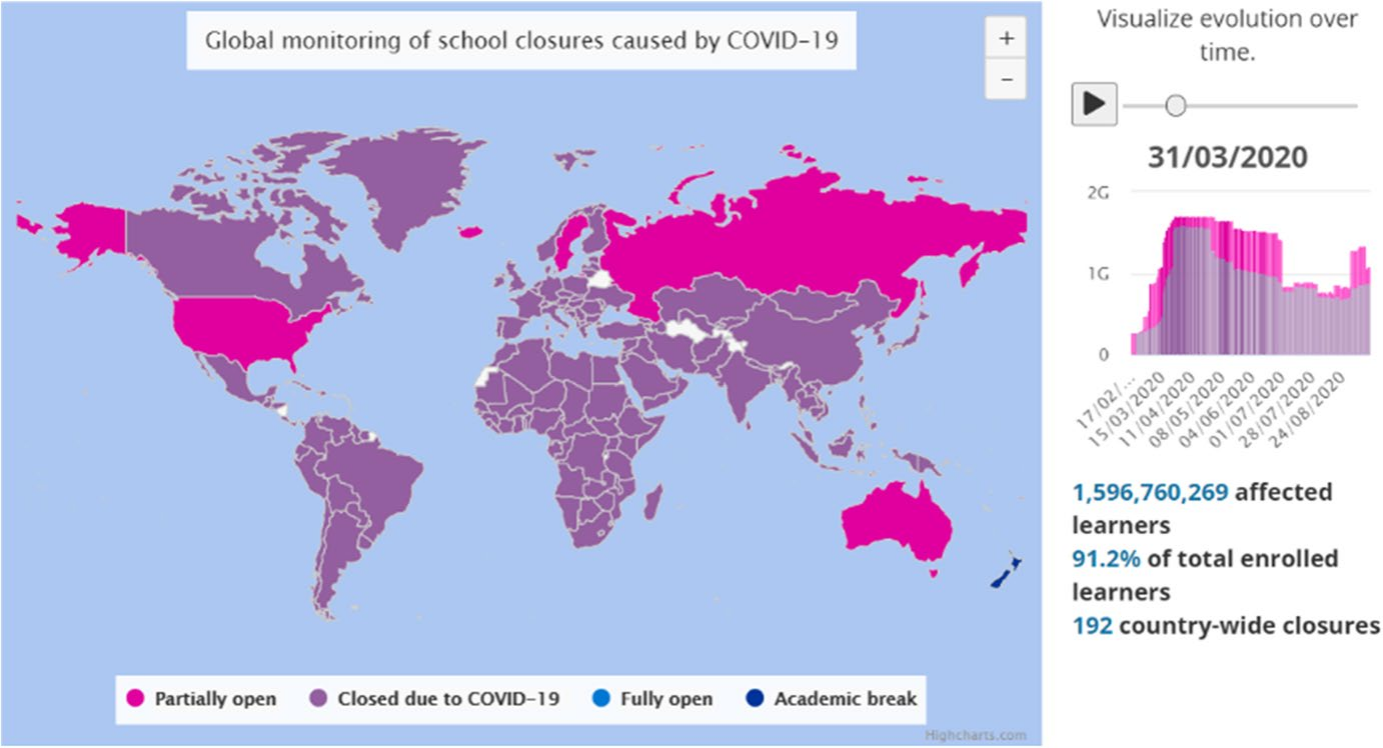
School closure in the world due to COVID-19 & affected learners: UNESCO. https://en.unesco.org/covid19/educationresponse

The global COVID-19 pandemic resulted in cancelling on-campus classes and forcing tertiary level students to shift to a complete online learning solution in a very short period of time (Milman, 2020). After the first shutdown of Chinese education system at the beginning of 2020 (Xiao & Li, 2020), soon most of the higher education institutions around the world chose to cancel all face-to-face classes including laboratories, libraries and sport activities (Aristovnik et al., 2020). UNESCO has estimated that 91.2% of the institutions and schools around the globe were closed by the end of March 2020 resulting from 192 countrywide closures. The data shows that (UNESCO, 2020) tertiary level students affected in Finland were 295,528 and in India 34,337,594 (see Fig. 1).

By mid-March, an emergency was declared by the World Health Organisation (WHO, 2020), and the lockdowns were implemented in both Finland (Finnish Government, 2020) and India (UN News, 2020) to prevent the spread of COVID-19 virus. It resulted in closure of universities and colleges in the middle of the Spring semester in Finland. The students were informed and immediately shifted to online learning methods. Though the countrywide lockdown restrictions were lifted after 8 weeks (UNESCO, 2020), most of the universities took the decision to continue a shutdown till date (University of Helsinki, 2021).^1^ Restricted use of laboratories started in May, allowing higher education students to attend practices in small groups. As it was the end of the semester during mid-March in India when lockdown was implemented, Indian universities and colleges followed separate patterns (IIT Bombay, 2020; St Xavier’s College, 2020)^1^ for adopting remote learning. Some universities immediately declared a preponed summer vacation only to start remote learning in June, whereas some colleges immediately shifted remote learning to complete the remaining curriculum till the end of that semester and continued the same after summer vacation in June. Face-to-face lectures were strictly suspended in India and no university started any physical session. Though remote learning started at the different time of the year in Finland and India, the students from both the countries shifted to an extraordinary learning experience called Emergency Remote Learning (ERL).

### Research on Emergency Remote Learning

When worldwide closers were declared, it resulted not only in prohibition of physical lectures but also closing libraries, laboratories, sports activities, changed communication channels and altered assessments (Aristovnik et al., 2020; Cao et al., 2020). The education delivered by the universities in the midst of the pandemic has a noticeable difference between conventional distance or online learning practices and sudden stopgap shifts as Emergency Remote Learning (ERL) was a temporary change for instruction and curriculum delivery due to COVID-19 crisis. Milman called it pandemic pedagogy (Milman, 2020) and described the situation as emergency remote teaching and learning, whereas Hodges created the term- Emergency Remote Teaching (Hodges et al., 2020) which has been interchangeably used as Emergency Remote Learning ERL by researchers (Rahiem, 2020; Schultz, 2020; Vollbrecht, 2020).

Several studies have investigated the unique education-in-pandemic situation. The research focusing on ERL has investigated different characteristics and trends of emergency remote learning. The researchers have noticed that the speedy transaction of online education during the pandemic was exceptionally fast (Schultz, 2020). Study done in British Columbia’s Okanagan campus in Canada (Riley & McNeil, 2020) has recorded that rapid transition to ERL had a negative impact on student’s mental well-being resulting in challenges in learning and engagement. Another study done in Indonesia (Rahiem, 2020) found that the students were not prepared for the transition resulting in paradoxical positive and negative responses about ERL. The study noted that the students were resilient and with the help of resourceful educators, they adopted blended methods which were the combination of video lectures, note taking, e-learning, and m-learning etc. A research on Geography education (Schultz, 2020) notes that emergency remote learning was unsatisfactory for students and teachers. It suggests the need for designing a well-structured course and its effective delivery.

### Higher education in the midst of pandemic

Many studies are performed centralising higher education during the pandemic. An extensive and comprehensive study (Aristovnik et al., 2020) on the impact of COVID-19 on the life of higher education students at the beginning of the pandemic investigated more than 30,000 participants across 62 countries. The study found that the students experienced boredom, anxiety, and frustration but the students showed engagement and improved performance. Another study found the challenges faced by Philippine’s higher education students were difficulties in adapting online activity, malfunctions of online platforms, and a poor internet connection (Edelhauser E., 2020).

The impact of pandemic on education in India and Finland was also reflected in country specific studies. A study done in India (Kapasia et al., 2020) reveals that the Indian higher education students noted problems related to anxiety, poor internet connectivity and unfavourable study environment at home.

Another study (Mishra et al., 2020) captured the insights of students and teachers who mentioned that it was a challenging experience in the sudden shifting scenario. Some of the key findings from this study are- free to access online educational resources, learner centred approach, systematic training to teachers etc. In their study on Indian higher education and research amidst pandemic, Rashid S. states practical problems like, vast digital inequality, lack of in-person teaching and struggle of students with no self-regulations, difficulty in seeking help, stress among teaching faculty, and limited sense of self belonging (Rashid & Yadav, 2020). Their study further suggests a development of e-content, assessments, and reporting. On the other hand, a focused study in medical education (Sahi et al., 2020) during pandemic suggests bringing technological advancements like virtual simulators, and video case vignettes to the forefront of online learning scenarios.

A very few studies are reported in English from Finland which focused on higher education in pandemic. But there are studies that focus on aspects of students’ experience. For example, Ranta’s study aims at knowing students’ personal concerns about mental wellbeing, studies, and economic situation (Ranta et al., 2020). The study found that young people are significantly more concerned about the impact of pandemic than older people in Finland. An international study on the effects of COVID-19 on mental health of adults noted that the pandemic had a lower positive and higher negative effect (Gloster, 2020) on Finnish people, although they reported higher levels of wellbeing. The study suggested the reasons can be social support, education level, finance, access to basic needs, and psychological flexibility.

### Student’s experiences, satisfaction, and engagement in online learning environment

In remote online learning, a technology mediation is required to transfer the skills and the knowledge to the learners. Hence, many factors in the form of experiences are important to make an online remote education effective such as conceptual understanding, interaction, technicalities, ease of access, and learner’s satisfaction is considered to be the essential element to measure quality of online learning (Rajabalee & Santally, 2020). Students’ satisfaction is the indicator of their overall educational experience and attainment. When implementing new technology or services, satisfaction is said to be the highly desirable outcome (Jung, 2014; Virtanen et al., 2017). A positive relationship between satisfaction and expectation was found in previous studies (Bailey & Pearson, 1983). In an educational setting, the level of students’ satisfaction can be the indicator of how well students adapt and accept a new technology (Ali et al., 2011). Usually, a survey questionnaire is used as a standard practice to measure student’s satisfaction, but there are other ways and tools which have been developed by the researchers. For example, Paul Ramsden’s Course Experience Questionnaire (Ramsden, 1991), the Research Initiative for Teaching Effectiveness (RITE) instrument which focuses on assessing learning engagement and interaction value which has been used by University of Central Florida (UCF) to survey online students since 1996 (Dziuban et al., 2015), and SSEQ (Students’ Evaluations of Educational Quality) instrument which evaluates students’ assessment on University teaching (Marsh, 1982).

An engagement is defined as the effort students put in the educational activities (Kuh, 2003) and it is considered as a key element to ensure quality and effectiveness of an online education (Rajabalee & Santally, 2020; Robinson & Hullinger, 2008). Swan defines three factors for a successful synchronous online learning- consistency in course design, interaction with teachers, and active participation (Swan et al., 2000). In general, a student is actively engaged by interacting, performing, attempting, thinking, and talking with classmates and teachers. Furthermore, an engagement is perceived as a student’s connection with the learning environment including behavioural, cognitive, and emotional aspects (Marx et al., 2016). The researchers have developed various tools to measure student engagement in online learning. Kuh’s (2003) National Survey of Student Engagement (NSSE) focuses on five activities including supportive environment, level of academic challenge, enriching experience, teacher and student interaction, and active learning (Robinson & Hullinger, 2008). The Student Course Engagement Questionnaire (SCEQ) developed by Handelsman et al. (2005) measures student’s opinion on attitude and behaviours. Similarly, Rrubric for Assessing Interactive Qualities of Distance Courses (RAIQDC) is designed to measure interactions by asking participants about other students’ behaviours (Robinson & Hullinger, 2008).

The Online Student Engagement (OSE) developed by Dixson (2015) considers SCEQ as a base measurement tool and adapts it for an online environment. It consists of 19 items grouped under Participation, Performance, Skills, and Emotion in online learning context. Dixon reports a strong significant correlation between active learning and higher engagement and supports student’s active engagement through participation and learning activities. It has been observed that an activity-based learning not only encourages student participation but also self-learning practices and higher cognitive skills. Students’ engagement is a crucial element which keeps students connected with the course and eventually their learning. Hence, it is highly important for an educator or educational researcher to effectively measure a student’s engagement.

## Research questions

The research filled the gap in knowledge (theory) of ERL with its practical implementation measuring by students’ satisfaction and engagement in the form of self-reported insights on challenges and experiences. The study focused on knowing how successful was ERL in different periods of pandemic for the students from different geographical location and whether it fulfilled learner’s needs. Further, based on the findings, the study also proposed guidelines for higher educational stakeholders to implement the most viable, sustainable, and valuable solution in the future.

Therefore, study aimed to explore research questions:

- What is emergency remote learning during COVID-19 crisis and how is it different from contemporary distance and online learning?
- What challenges and experiences were common and country specific among university level students from different geographical backgrounds?
- What are the effects of the long-term use of ERL?
- What impacts on student learning engagement resulted from ERL during COVID-19 pandemic?
- What guidelines for better learning experience can be derived from the study findings?

## Theoretical model

**Fig. 2:**
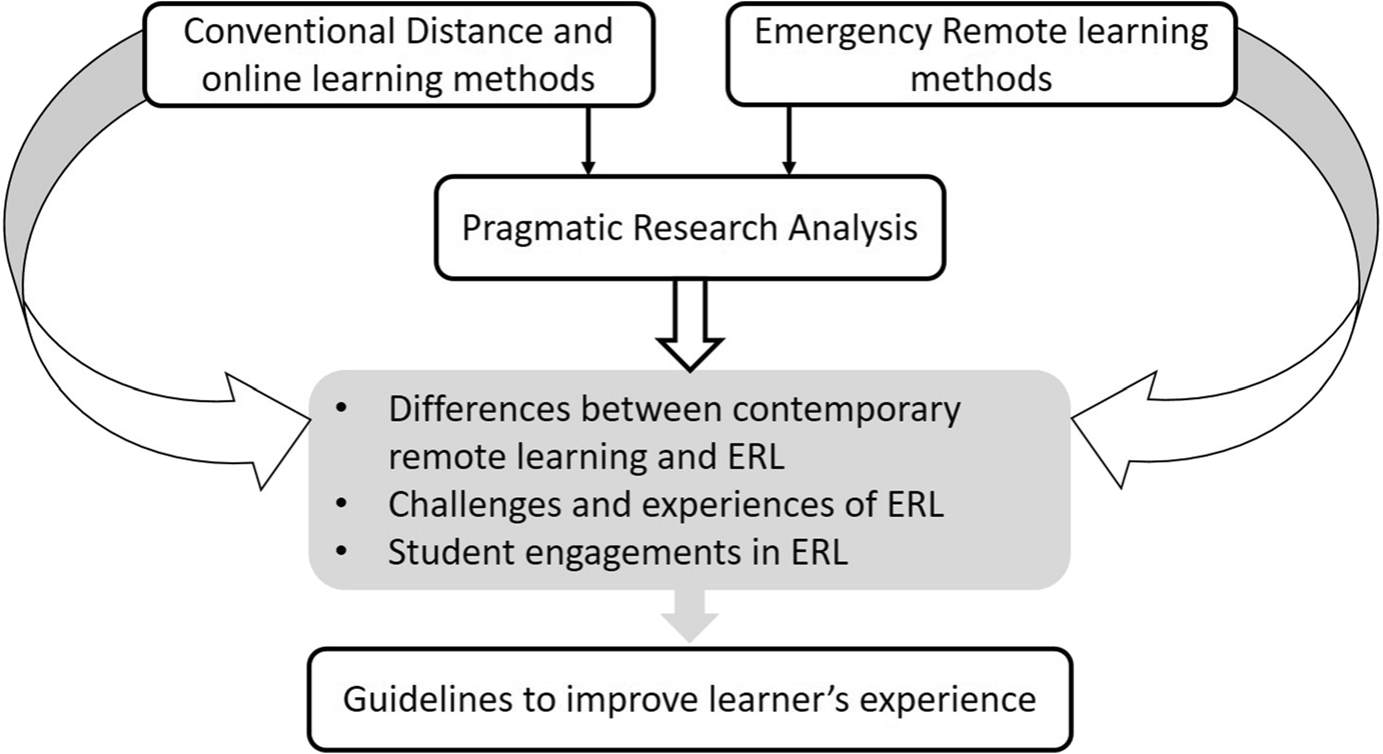
Theoretical model used in the study

The authors investigated the research questions in a twofold study. The first step towards understanding the ERL learning was to review the definitions and meanings of different remote learning solutions in practice. It resulted in defining similarities and differences between ERL and other options. The authors also investigated the different learning platforms that are getting utilized by current practices in contrast to ERL platforms. Therefore (see Fig. 2), the theoretical model started with a comparative analysis of ERL and all other conventional remote learning methods. The analysis included establishing similarities and differences between them to define characteristics of each one separately. Based on these findings, the second part of the study, as shown in the model, was to conduct pragmatic mix-method study by investigating surveys, diary study, and semi-structured interviews. The analysis of these studies gathered the insights on challenges, experiences, and student engagement in ERL. These findings are collated together and analysed with the characteristics of ERL defined in the first part of the study to develop guidelines and recommendations to improve current ERL features and its implementations in the future.

### Part one: conventional and emergency remote learning solutions

To an observer, various remote learning methods- distance education, online learning, or emergency remote learning seem like a trivial distinction to make. But for the education professional and researchers these specific terms are critical as they represent different methods of learning (see Table 1). The importance of knowing the right method is magnified when referencing different types of learning methods can lead to distinct directions which can or cannot be relevant to the current challenges.

### Distance learning

Distance learning as the name suggests, means learning from a distance when the teacher and students are in different geographical locations. Originally it was introduced as correspondence courses and the exchanges between learners and teachers happened through postal services (Stafford Global, 2020). It is self-paced learning with instructor facilitation, from different locations and time. Expansion of the internet has expanded distance learning to online learning. Though many well-known universities offer distance degrees or courses, some ‘Open Universities’ also provides the education with minimal or no entry requirements. Examples of open universities include The Open University, Trinity college of Dublin, Kent University, and Indira Gandhi National Open University (IGNU) etc. (Wikipedia, 2020). The universities mostly use their own web-based software or platforms to facilitate classes (Stanford Online, 2021). Some universities affiliated with governments broadcast their classes on educational television channels, such as Gynadarshan (IGNU, 2021). Distance learning offers both synchronous and asynchronous (see Table 1) mode of learning and is facilitated by the instructors (Berg, 2016). Main advantage of distant learning is that it is offered with lesser fees compared to mainstream universities and the degrees/credits obtained from distance learning are mostly accredited. Generally, adults returning to higher education, students who live in remote places update their knowledge using distance education or students who cannot afford university education choose distance learning options.

**Table 1:** List of conventional remote learning methods along with ERL and their characteristics

Method	Physical/remote	Interaction	Intention
Distance learning	Remote	Asynchronous or synchronous	Self-paced, curriculum designed for students from different geographical areas
Online learning	Remote	Synchronous or asynchronous	Topic specific courses selected by students mainly to upskill in particular topic
E-learning	Remote	Synchronous or asynchronous	Timebound, specific in-depth knowledge
Virtual learning	Physical + anywhere	Asynchronous	Students have remote access to instructor and content
Blended learning	Physical + remote	Asynchronous and synchronous	Blend of face-to-face learning and virtual learning
Emergency Remote Learning	Remote	Synchronous	Makeshift in the crisis, delivering mainstream curriculum

### Virtual learning, e-learning, blended learning

Variety of terms that are connected to distance learning have evolved, describing the different ways in which one can pursue a degree or attend a course remotely, namely online learning, e-learning, or blended learning. Virtual learning delivered (Beek, 2011) through internet, software or both can be conducted inside or outside the physical building of the educational organization and can be blended with face-to-face learning by including videos, podcasts, screen captures, animations for PowerPoint presentations. Virtual learning method is usually chosen by the instructors and used as part of the main curriculum. Students have remote access to the course contents and instructor. In E-learning or Electronic learning on the other hand, the students can only interact with the instructors through online medium and have limited access to the content (Shahabadi & Uplane, 2015). E-Learning is used for teaching specific skills or in-depth knowledge in a structured, formal, and time-bound manner. The students can choose to take up an e-learning course to upskill or learn apart from their regular college curriculum. Blended learning is a combination of distance learning and traditional on-campus. Blended learning can be part of an entire curriculum of distance education or can be chosen by an instructor to achieve individualised learning experience.

### Online learning

Online learning involves internet connection and mostly a synchronous learning experience (see Table 1) in the form of webinar, online lecture, or a virtual meeting. These voluntary courses may have more choices when it comes to course selection (Stafford Global, 2020). Online courses offered by well-known universities like Stanford (Stanford Online, 2021) conduct them on their own platforms. The courses can be free or paid and recognised globally. Professional courses offered by companies have more focused curriculums like Microsoft Learn (Microsoft products related), Google Garage (Digital marketing), Pluralsight (developer skills), and W3Schools (Webpage development) etc. The courses offered by universities or private contributors use their own digital platforms to facilitate learning and include blended experiences for assessments like quiz, problem-solving case studies, blogs, or online tests. Online learning has gone to a different level with the immersive platforms offered by universities (Digital Initiative, 2021), or private contributors (Second Life, 2020) which give enhanced participant presence in these courses.

Massive Open Online courses (MOOCs) diversely, are more contextualised open platforms which collaborate with various universities and private contributors to facilitate mostly free or cheap online education for a larger number of students who want to upskill for employment or knowledge (Pouezevara & Horn, 2016). MOOCs usually offer asynchronous learning where the instructors do not see and interact with students, do not have personalised experience, and the courses are conducted in the specific duration where the participants (larger group) are assessed by group assignments and/or discussions in online chat with all the participants. In this complete online method, the students may or may not have the channels like blogs, chat rooms etc. to interact with each other. University owned MOOCs (MOOC.fi, 2021; Stanford online, 2021) offer direct credits whereas certificates issued by private contributors like Coursera, Udemy, edX etc. hold value in the job market, but its valuation varies in different countries. MOOC courses if taken outside of the course duration, might not facilitate certification or credits at the end. Though many MOOCs platforms are free, private contributors follow different payment structures like cross subsidization or monthly subscriptions etc.

In all these forms of distance and online learning involve significantly more planning and design and ideally inputs from instructional designers. The curriculum is usually planned separately to fit the need for distance learning methods. The expected communication during the synchronous and asynchronous methods are carefully planned, so the students can make most of the course content and be satisfied with the learning experience. For a fully online university course typical planning, preparation, and development ranges from 2 to 9 months (Hodges et al., 2020).

### Why is Emergency remote learning different?

Emergency Remote Learning has emerged as a common alternative term used by online education researchers and practitioners to draw similarities and contrast with high-quality online learning (see Table 1). Because of COVID crisis, the institutions are trying to fill the gap of face-to-face education by creating an online synchronous experience for the entire curriculum. Instead of conventional structural design of the courses (Hodges et al., 2020), the content is being converted hurriedly into virtual experience using PowerPoint presentations, videos, and screen captures (Aristovnik et al., 2020; Schultz, 2020). Students who have enrolled for regular university degree education were not asked to enrol for ERLs separately. Because of the COVID-19 crisis, it is not a student’s choice whether to take or not these ERL courses offered by their universities but has merely become a compulsory method to complete their degree education failing which can result in serious effects on their educational career. Students are completing assignments, group projects, and even giving exams using ERL methods with which their performance is getting recorded similar to regular face-to-face education.

Another principal difference between the conventional methods and ERL is the time duration expected to spend on learning. The students are expected to attend ERL like face-to-face education where the students spend their entire day-approximately 5 to 6 hours attending lectures, whereas in the voluntary conventional method, students are not expected to spend more than planned or allocated time, usually 2 hours a day. The contrast between conventional methods and ERL is that ERL is mostly used for synchronous education and is conducted on 3rd party digital resources like (Rahiem, 2020) Zoom, Microsoft Teams, Webex, and Google Classrooms instead of conventional structured MOOC or online platforms. The students are also using these platforms for presenting their work, asking questions, and communicating with instructors or classmates. These instructors are university professors who are used to teaching face-to-face or blended methods. Most of the instructors are neither trained to teach their curriculum using new online platforms nor are they prepared for instructing longer hours or assessing students completely virtually (see Fig. 2). On the other hand, without the help of instructional designers or defined structures, instructors are finding it challenging to convert the entire curriculum to fit the needs of third-party online platforms.

In the rush job to provide synchronous online education, ERL is facing some major issues like security threats for example, Zoom Bombing (O’Flaherty, 2020) and lacking well-planned blended methods which are usually taken care by conventional online learning platforms.

### Part two: challenges, experiences, and student engagement in ERL

**Fig. 3:**
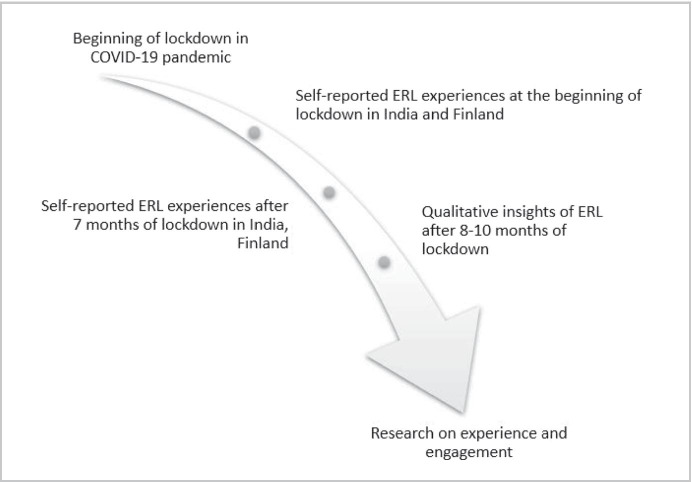
Longitudinal data triangulation shows for the study

The second part of the study (see Fig. 2) delves into the experiences of the students from higher education and their own interpretation of challenges and learning engagements in ERL during COVID-19 crisis. To explore the research questions, the study is grounded in the pragmatic approach and was carried out using a mixed research method comprising surveys, semi-structured interviews, and diary study. The study was done in the unique situation of pandemic required a detailed and balanced picture of students’ insights on ERL and their learning engagement (Robinson C., 2008). Hence, more attention is paid to data triangulation (see Fig. 3) to map out complex behaviour of the students by studying it from more than one standpoint (Cohen et al., 2017). The population sampling targeted students from higher education with at least 18 years of age. The participants were recruited using convenience sampling facilitated by advertising on university communication systems, social media as well as authors’ tie-ups and contacts separately in Finland and India.

### Materials and methods

The study was initiated by conducting a survey in two stages. This longitudinal approach (see Fig. 3) was taken to capture the rich insights of ERL from the different stages of the pandemic and to investigate the long-term impact of ERL. Hence, the first survey was conducted from 19th May to 7th June 2020, during the beginning of the pandemic lockdown where the students were new to ERL and were studying from home for the period of a month depending on university specific curriculum and country-specific lockdown decisions. The same survey was then conducted again from 21st October to 16th November with a new set of students when they had experienced ERL for at least a complete semester (5 to 8 months), depending on country and university specific decisions and when the students got used to the ERL setup. The survey was published in the internal communication network of universities in Finland through an article to reach out to the students from all disciplines. Similarly, the author contacted a number of institutions and universities from India asking their permission to survey their students. The open call for the survey was also posted on social media channels like, LinkedIn and Facebook.

There were multiple reasons why researchers chose the method of convenience sampling and logitudinal approach. The first reason was the ease of access of the university level students which was restricted due to COVID-19 situation. Second, including diverse data from developed and developing country backgrounds helped the study to get a more comprehensive picture of common experiences instead of cross-sectional data of a particular region. Third, due to COVID-19 situation most of the universities and institutions from targeted countries were shut down from March 2020 (UNESCO, 2020) and during the survey investigation (May 2020–January 2021) the physical campuses remained closed, the study could capture the wider spectrum of pandemic stages. This situation helped gathering data for different components like challenges, engagments etc. Lastly, since the students were not recruited from a course/university or discipline, the completion of the survey and diary study varied in both the countries depending on voluntary participation.

On the other hand, the semi-structured narrative interviews and the diary study were conducted later in the pandemic (After 8–10 months of lockdown) to capture more insightful impact of ERL with the participants of the first survey and the second survey who had shared their interest in the further study. They were contacted in mid-October and November 2020, respectively. The synchronous interviews were conducted over Zoom, using only audio, with individual participants. Whereas the diary study was administered from the last week of October 2020 to the third week of January 2021 depending on the availability, class schedules, and convenience of the participants.

### Measures

This mix-method study started with a web-based short survey questionnaire distributed twice in the different stages of pandemic lockdown, followed by 30 to 40 minutes of online interview, conducted with each participant separately. The last part was a diary study carried out using Microsoft Forms for the period of 5 days.

For the survey design, a customized questionnaire was used as a main tool to record student's experiences to know their satisfaction towards ERL (Ali et al., 2011; Rajabalee & Santally, 2020; Virtanen et al., 2017). To achieve face and content validity, the survey questionnaire was checked by two experts in the field (university professor and Postdoc researcher). The survey was also peer-reviewed by three researchers. Furthermore, to assess the reliability of the questions and their internal consistency was calculated using Cronbach’s Alpha for set of related questions separately.

The survey questionnaire composed of 15 mixed questions. Refer to Online Appendix A for survey sections and list of questions. The first section comprised five questions on demographics and academic backgrounds of the students, e.g., country, level of study, age group, gender, and discipline. The second section contained four questions related to ‘Online experience’ and the first question was about their overall remote experience which was composed of a five-point Likert scale where 1 = love it and 5 = terrible, following with questions on duration of ‘online learning’ per week, study topics (open ended) and assessment types (multiple choice question).

The third and the last section was focused on ‘challenges and learning experiences of ERL’ containing total seven questions. A multiple-choice question on challenges (Online Appendix A, Section 3) was composed of 11 statements including a statement if ‘no challenges’ were faced. Hence an average variable ‘total challenges’ was computed based on 10 multiple choice statements. Descriptive statistics of original statements on challenges are displayed in Online Appendix B1. An average variable, named ‘total challenges’ was calculated based on the individual items. The reliability of the scale was moderate (α = 0.637). The variable was not standardized hence the mean for all questions is 4.23 and the standard deviation is 2.22 (see Table 3).

The next question on benefits of ERL composed of 7 items. All items were measured on five-point Likert scale where 1 = Extremely and 5 = Not at all. An average variable was calculated based on the individual questions on benefits. The reliability of the scale was good (α = 0.820). The variable was not standardized so the mean for all questions is 3.13 and the standard deviation is 0.90 (Online Appendix B3). The question on quality of learning was composed of a five-point Likert scale where 1 = not satisfied and 5 = extremely satisfied. The questions on interactive experience of ERL consisted 5 items and all items were measured on Likert scale where 1 = Very interactive and 5 = Not at all. The participants were asked about their willingness to continue remote learning post-COVID using a five-point Likert scale where 1 = full time online and 5 = no online at all, only in-person. The participants were also asked about their expectations from future solutions using open ended question.

On the other hand, as the next part of the study, synchronous semi-structured interview questions consisted of four open ended questions succeeding with contextual tail questions focused on participant’s details about current curriculum, ERL tool/s used for learning, and his/her overall experience for the past 6 months. One of the questions was focused on understanding key demographic details establishing students’ study logistics, and pre-pandemic experiences with remote learning to gather the data on the familiarity with the conventional remote learning methods. In synchronous interviews the participants were asked to respond and elaborate upon semi-structured prompts (Online Appendix C).

Diary study was designed using the Online Student Engagement (OSE) questionnaire (Dixson, 2015) to gather data on learning engagement of ERL. However, an open-ended questionnaire was created based on OSE factors- Skills, Performance, Participation, and Emotion (Online Appendix D) for capturing in-depth qualitative data and the participants shared their insights in the form of short answers. A constraint was recorded for the modification of question delivery. The original OSE instrument perceives students’ self-report through survey questionnaires but the current study altered it to capture qualitative insights.

Diary study was conducted for one week or five consecutive days using a web-based form. Just before starting the study, one of the authors had a 30-min online meeting (only audio) with each participant to explain the design of the questions, expected actions from the participants, and a brief on use of the form. Throughout the study, the participants were supported through email communications and the authors kept a close attention to the inputs received from participants each day. During the study, every evening, the participants shared their inputs after attending all classes or course meetings for the day.

The diary form comprised two main sections (Online Appendix D). The first section focused on the overall learning experience of a participant for that day whereas, the second section aimed at gathering qualitative information of a particular online class the participant attended during that day. If the participant attended more than one online class during that day, the questions of the second section were repeated in consecutive sections to collect data for those classes separately. As a result, the number of the sections in a form were contextual for each participant and depended on the number of classes the participant attended each day (see Table 2) and the number of diary entries of each participant varied depending on the number of classes he/she attended. The information about the number of classes each participant will attend, during 5 days of diary study, was recorded during the briefing meeting and the form was modified for each participant in advance. Another online briefing meeting with some participants was conducted (audio only) for 10 to 15 minutes 1 or 2 days ahead of the diary study to address doubts and questions. The research was carried out in accordance with the Responsible Conduct of Research (RCR) guidelines of the Ethics Committee Tampere and Finnish National Board on Research Integrity (Ethics Committee Tampere, 2010; TENK, 2012) and the participants’ information kept strictly confidential and anonymised for data processing.

**Table 2:** Demographic, geographic data of diary study participants with classes attended every day

Participant ID	Country	Gender	Discipline	Classes every day
1	Finland	Male	Electrical engineering	1
2	India	Female	Management studies (BMS)	6
3	India	Female	Management studies (BMS)	5
4	Finland	Male	Pedagogy	1
5	India	Female	Sociology	2
6	Finland	Female	Game Studies	2
7	Finland	Female	Environmental Engineering	3

The first section confined four closed ended quantitative questions- “rate today’s overall learning experience for all classes” (5-point rRating), “how was your overall performance” (5-point Likert scale where 1 = very bad and 5 = extremely well), “how long did you attend online classes combining all” and probed participants on their emotional reflection using 5-point Likert scale where 1 = sad/depress/down and 5 = happy/excited/awesome/great. The second section consisted of eight open-ended questions manifested main behavioural components of learning engagement- skills, performance, participation, and emotion. In this section the participants were also explicitly asked to record daily learning challenges and aspirations. The participants also rated (5-point scale) the experience with their course teacher and recorded their interactions during the class like, whether their video was on, could they see teacher, and whether they were distracted by doing other things during the class using a 5-point Likert scale where 1 = Never and 5 = All the time.

### Statistical methods

The study used IBM SPSS Statistics 26.0 software for data management and to analyse quantitative data. Microsoft Excel 360 MSO Version 2012 software was used to analyse qualitative data. Quantitative data gathered from the survey 1 and 2 was categorised in accordance with the research questions. These categories were carried forward to analyse, find patterns, and run statistical tests.

The quantitative study focuses on challenges and experiences faced by participants while attending ERL. Challenges and other specific variables/indicators which reflected students’ experiences such as overall learning experience, satisfaction towards learning quality, post-COVID remote learning preference, and benefits are analysed to probe quantitative results. To test differences between Finnish and Indian students and selected variables/indicators of student’s experiences, statistical T-test, or Mann–Whitney U-test was used after checking normality of the distribution. Mann–Whitney U-test and T-test is also used to test differences between short term and long-term effects of ERL depending on the normality of the distribution (Online Appendix B2). Additionally, Spearman’s correlation test is used to test statistical dependency between challenges and other experience indicators such as, overall learning experience, learning quality, and post-COVID remote learning preference. Participants who didn’t report any challenges (*n* = 6) were omitted from this comparison. One participant who didn’t give input on ‘learning quality’ was also omitted while comparing it with challenges (*n* = 131).

To check the normality of the data Shapiro–Wilk test was used with null hypothesis indicating data is equally distributed and normal. The distribution of total challenges among participants has skewness of 0.504 (SE = 0.211) and a kurtosis of -0.110 (SE = 0.419) indicating that data is little kurtotic. Shapiro–Wilk test results shows that the data is statistically significantly differ from normality W (132) = 0.948, *p* < 0.001 hence, null hypothesis is rejected indicating the data is non-normal. (Online Appendix B2). Similarly, the distribution of ‘overall remote learning experience’, ‘learning quality’, and ‘post-COVID remote learning preference’ were non-normal, hence Mann–Whitney U-test is used for statistical comparison. However, the data of ‘benefits’ is normally distributed, hence t-test is used for statistical comparison (Online Appendix B2).

On the other hand, affinity process (Nowell et al., 2017; Rosala, 2019), a form of thematic analysis, was used to probe qualitative data derived from the diary study and the semi-structured interviews. An affinity diagram (Dam R.F., 2020), a tool to conduct affinity processes, was used to organize qualitative data and to create groups based on their natural relationships. Affinity map helped to collate similar findings into groups and label them based on their themes, instead of conventional approach where the data is labelled based on the pre-designed categories. This bottom-up approach not only helped to derive significant and obtrusive themes, but also helped to find unique patterns in the data. We anonymised each individual's data and coded every participant as S1 to S138 for surveys, D1 to D7 for diary study and IN1 to IN7 for interview analysis. Moreover, for each statement we added a date stamp (mmddyy) as a code. For example, a statement quoted by participant 7 in a diary study on 18th November 2020 is displayed as D7:111820.

### Participants

The study targeted university level students (first cycle bachelor to third cycle doctoral degree students), age varying from 18 to 45 and above, from two different geographic locations, who were regularly attending classes from home, were learning core degree curriculum as per university specification and experiencing ERL for an extended period of time (10 months). Total 138 students from different disciplines participated in the study and overall participants from each country had an unequal distribution as follows: Finland 29% (*n* = 40), India 71% (*n* = 98). The 39.13% (*n* = 54) participants were male, 57.25% (*n* = 79) participants were female and four preferred not to disclose their gender and one participant was non-binary.

By June 2020, after 19 days of open call, 62 students participated in the first survey—Finland (29%, *n* = 18), India (71%, *n* = 44). The 50% (*n* = 31) participants were male, 47% (*n* = 29) were female and two preferred not to disclose their gender.

Conversely, for the second survey, after 26 days of open call, by mid November 2020, a total 76 students participated from Finland (28.95%, *n* = 22) and India (71.1%, *n* = 54). The 30.3% (*n* = 23) participants were male whereas, 65.8% (*n* = 50) participants were females, two participants preferred not to disclose gender and one participant was non-binary.

Semi-structured interviews and diary study were conducted with the same participants who voluntarily shared their contact ID through surveys, agreeing to be part of the further study. 30 out of 62 participants from the first survey and 40 out of 76 participants from the second survey were contacted. Finally, a total of seven students from Finland (57%, *n* = 4) and India (43%, *n* = 3) participated in the individual online interviews (see Table 2). The same students also participated and completed diary study. In the diary study, each individual shared qualitative insight for the duration of 5 days. Two were males and five (71%) were females. Interview data of two participants were dis-qualified and have not been considered in the data as they did not appear for dairy study.

### Ethical considerations

The study followed the GDPR regulations (EU-GDPR, 2016/679) for data collection and process in Finland as well as in India.^2^ The study also followed the ethical guidelines of the Finnish National Board on Research Integrity (TENK, 2012) and Ethics Committee Tampere (Ethics Committee Tampere, 2010).

An informed consent was shared with survey participants at the start of the form. Participation was anonymous except in one question where the students voluntarily shared their email ID to participate in the interviews and the diary study. Participants of the qualitative study again gave an oral consent at the beginning of the semi-structured interviews. The consent was also repeated during the informative meeting for the diary study. Participation in all three stages (survey, interview, and diary study) of the study was voluntary and students were informed that they can withdraw their participation at any point without any consequences. The survey was shared with students from higher education institutes from Finland and India. To follow the ethical data collection guidelines of ethical review in human sciences (TENK, 2012) the survey was shared with people aged 18 and above. The authors also had planned to remove the data of participants below 18 years, but no such case was recorded. Only the authors had the access to the research data, and it was fully anatomised before processing.

### Results

The results revealed that the learning experience of the students from both developed and developing countries was not satisfactory. They noted the struggle to get adjusted with the pandemic situation, new learning environment, and change in daily schedule. The research found out that the students from a developing country like India faced similar challenges to the student from a developed and technological advanced country like Finland (OSF, 2020), such as managing schedules, experiencing boredom, distractions, and pessimistic emotions towards ERL. They showed moderate learning engagement. Eventually, no long-term effect of ERL was found, as the students started adjusting to the new normal, but they didn’t indicate the complete acceptance of ERL.

#### Challenges in ERL

**Fig. 4:**
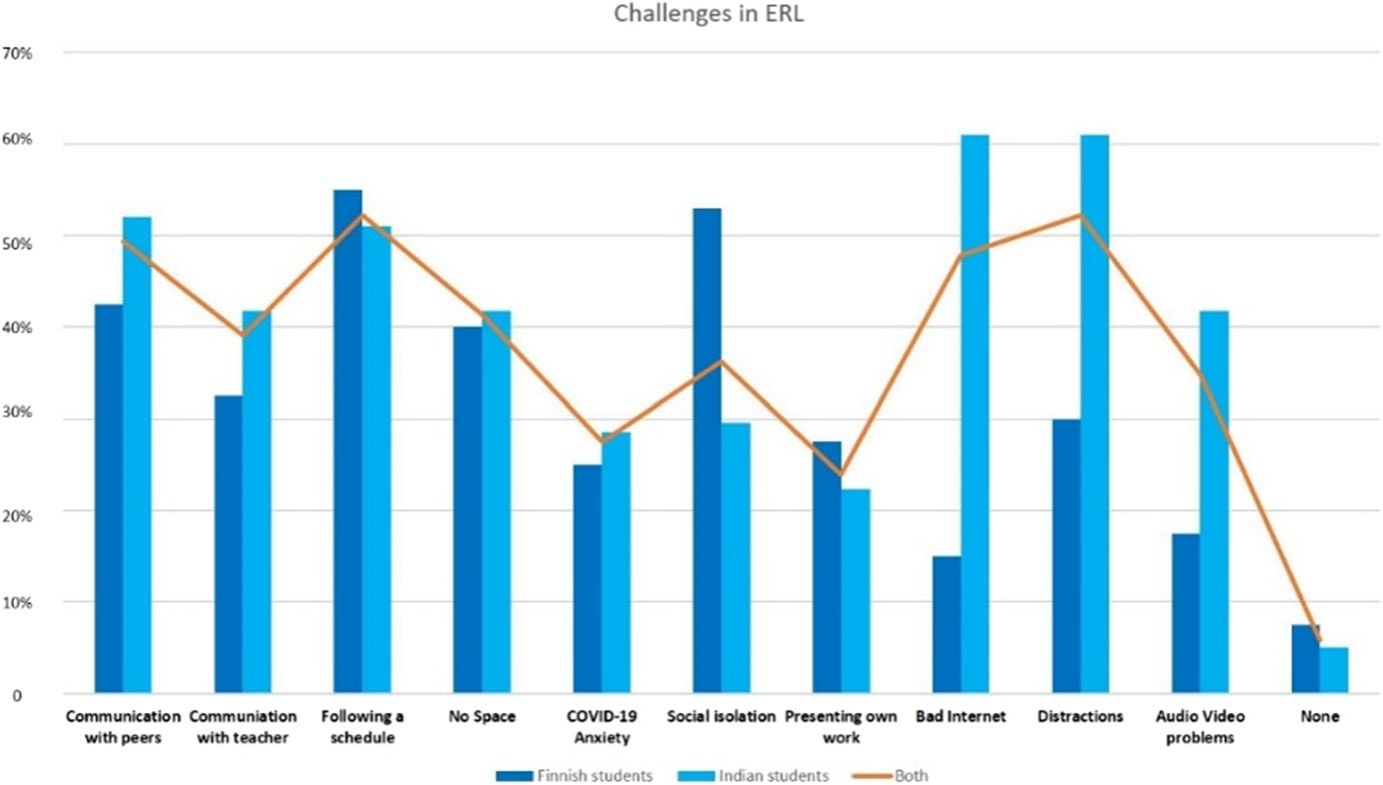
Total and separate % of challenges reported in both surveys by Finnish and Indian students

132 participants (95.65%) out of 138 from both the surveys faced at least four (*M* = 4.23, *SD* = 2.22) challenges with ERL (Online Appendix B1). The challenges which are mentioned by most of the students were (see Fig. 4) managing a schedule (52%, *n* = 72) and distractions in home-learning set up (52%, *n* = 72). The students also faced difficulties in communicating with peers (49.3%, *n* = 68), bad internet (47.80%, *n* = 66) and space issues at home (41%, *n* = 57). Surprisingly, very few participants (27.5%, *n* = 38) reported COVID-19 related anxiety and only six students out of 138 (4.3%) reported that they did not face any challenges.

Country specific data shows that 95.92% (94 out of 98) Indian and 95% (38 out of 40) Finnish students faced at least 4 different type of challenges with ERL (see Table 3). Indian students reported higher number of challenges (*Mdn* = 4) than Finnish students (*Mdn* = 3.50). A Mann–Whitney U-test indicates that this difference was significant *U* (Indian students = 94, Finnish students = 38) = 1331.00, *z* = -2.31, *p* < 0.05, as Indian students were more distracted (61%, *n* = 60), had issues with internet connectivity (61%, *n* = 60), faced problems communicating with their peers (52%, *n* = 51), and had difficulties keeping a consistent schedule (52%, *n* = 51). Similarly, Finnish students found it difficult to manage a schedule (55%, *n* = 22), but felt more isolated (53%, *n* = 21) than Indian students (see Fig. 4).

**Table 3:** Total and separate % of challenges reported in both surveys by Finnish and Indian students

Descriptive statistics of challenges						
Variable	Mean	SD	95% Confidence interval for mean		Skewness	Kurtosis
			Lower bound	Upper bound		
Total challenges	4.23	2.22	3.85	4.61	0.504	− 0.11
Finnish participants	3.55	2.3	2.8	4.31	0.754	0.174
Indian participants	4.5	2.14	4.06	4.94	0.502	− 0.021

*SD* standard deviation

##### Qualitative findings

**Fig. 5:**
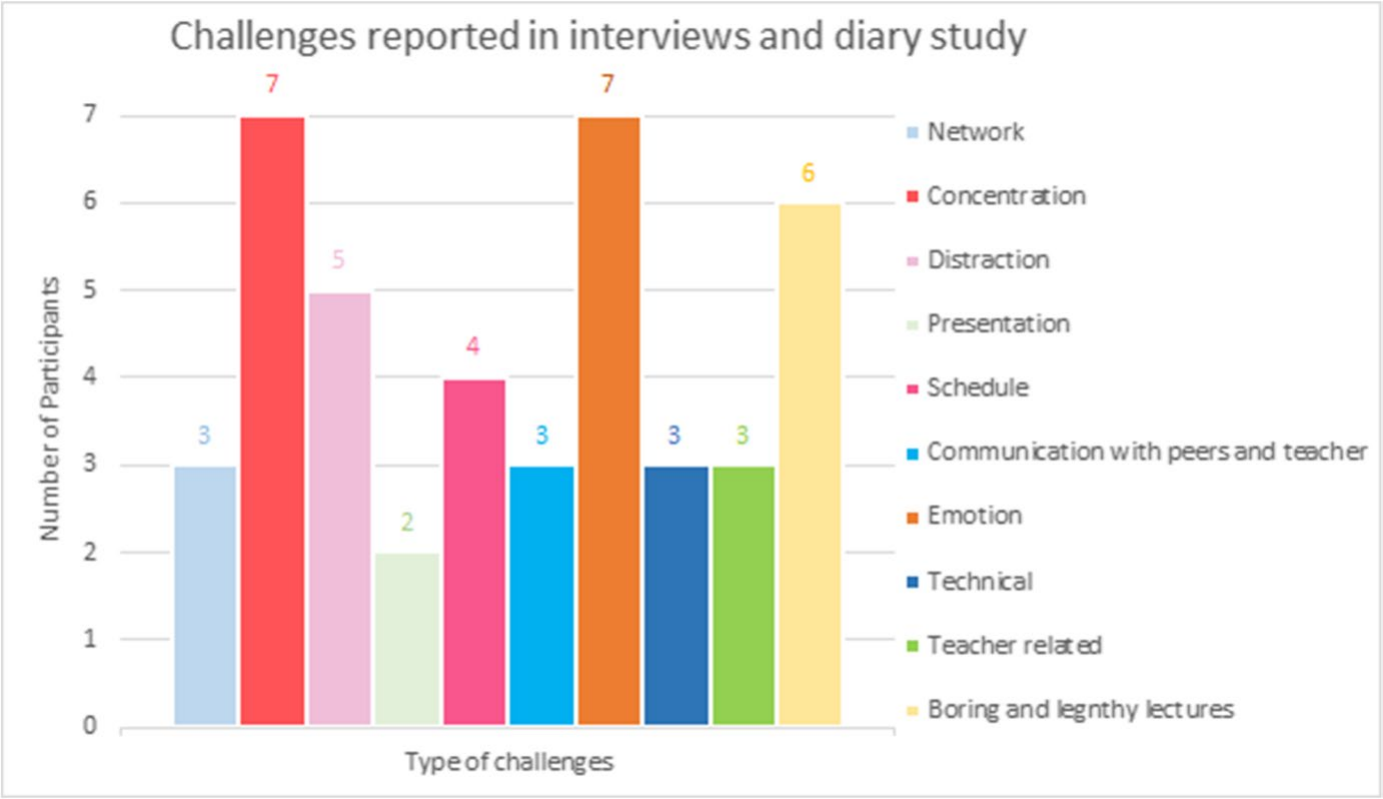
Qualitative data on challenges reported by participants in interviews and diary study

Qualitative data derived from diary study and semi-structured interviews also shows that all participants (*n* = 7) from both countries faced various challenges (see Fig. 5) while using the Emergency Remote Learning method. Each participant reported at least five different categories of challenges (*M* = 5.86, *SD* = 1.35). One of the most critical challenges (100%, *n* = 7) reported was difficulties in concentrating during the lectures. The reasons behind poor concentration were related to the non-interactive communication and extreme comforting home-setups.

“My focus drifted away from time to time, which always happens during Zoom lectures (I can't remained focused for long in online environments)”. (D6:11221)

“I found it hard to concentrate. We had this session right after lunch. I was sleepy. Moreover, I attended this class from my bed and not my study table. It was far too comfortable”. (D5:110520)

The other key challenge mentioned by all participants (100%, *n* = 7) was related to pessimistic emotions. One of the reasons behind ‘not feeling like studying’ during ERL lectures was monotonous ERL experience which affected their motivation.

“Can’t press the refresh button. It has started becoming monotonous around. No break from the same environment”. (IN5:102420)

“I don't feel like studying, so I take notes from other classmates. When I feel studying then I attend the whole lecture”. (IN3:113020)

Participants also complained about (see Fig. 5) long duration (81%, *n* = 6) and distractions (71%, *n* = 5). The students were not happy when the lectures lasted longer than an hour or when they attended back-to-back many classes (see Table 2) in a day. They noted that to accommodate regular curriculum, the duration of the classes/lectures were either extended or conducted based on the regular timetable. On the other hand, distractions like interruptions by family members, social media posts, sound disturbance, food or games were described by the participants.

“Listening to a 2 h monologue and watching the slides of a PowerPoint is not very effective for an online class”. (D7:011721)

“Time of my classes changed and the teacher kept on extending it. I could not take it anymore. So last week I made a point and did not attend that class”. (IN5:102420)

“I was distracted because everything was so funny. We have our WhatsApp group- So for quite some time, we were sharing funny gifs and pictures on our informal group and laughing about it”. (D5:111020)

Country specific qualitative data shows that, all Indian participants experienced bad internet connection and mentioned the problem many times (40% of the days) during the 5 days of diary study. They also reported boredom, as they attended synchronous learning and more classes (see Table 2) per day (*M* = 4.33, *SD* = 2.08) than Finnish students (*M* = 1.50, *SD* = 0.58).

“Sometimes because of network issues ya..it is difficult to attend classes”. (IN2:120120)

Oppositely, only Finnish students (75%, 3 out of 4) shared technical difficulties and challenges related to home-learning arrangements, like lack of required software or hardware (see Fig. 4). Finnish students attended both synchronous and asynchronous learning with combinations of lectures and self-study using materials shared on the university’s MOOC. 50% (*n* = 2) of them were disappointed when some teachers completely skipped the ‘teaching’ part of the course and assessed their knowledge based on the self-study and no changes were made in the curriculum or course syllabus for ERL experience. Hence, though they gave average ratings (*M* = 3.06, *SD* = 0.78) to their experiences with teachers during diary study, Finnish students (75%, *n* = 3) also reported teacher related challenges.

“I did face a big challenge after the lecture when I realized that a program we will have to use is not compatible with my PC. There is an individual assignment that we have to complete by using digital tools only and unfortunately my technology doesn't support them. So I am quite sad/nervous that I will maybe have to drop the course because of this”. (D6:011121)

“Teachers say every now and then or every second class that we don't have time for this class today so here is the material you can read about or here is the assignment”. (IN7:113020)

#### Experiences of ERL

##### Benefits

Though no prominent benefits of ERL are reported in the surveys (Online Appendix B3 and B6), interviews or diary study, one of the most mentioned of all was ‘no travel time’. 37% (*n* = 51) students found that ERL extremely helped them to save travel time (*M* = 2.59, *SD* = 1.58). Students also liked the fact that they can join the classes from the comfort of their home 23% (*n* = 32). Country specific data indicates that Indian students appreciated (38%, *n* = 37 out of 98, *M* = 2.70, *SD* = 1.66) the fact of reduced travel time due to ERL more than the Finnish students (35%, *n* = 14 out of 40, *M* = 2.33, *SD* = 1.35). But Finnish students (30%, *n* = 12, *M* = 2.51, *SD* = 1.37) enjoyed the comfort of home more than the Indian students (20.4%, *n* = 20, *M* = 2.58, *SD* = 1.11). Qualitative findings show that only Finnish students (50%, *n* = 2 out of 4) noted that remote learning is the way forward and they were used to technology advancements and connecting online remotely. But there are no significant benefits noted by Indian students (*M* = 3.19, *SD* = 0.82) than Finnish students (*M* = 3, *SD* = 1.09) in a t-test, *t* (136) = -1.11, *p* >  0.05).

Corresponding qualitative data collected in diary study and interviews (Online Appendix C and D) also echoed the findings, as students reported saving 2–3 hours daily in traveling especially during winter and the comfortability of getting up late due to saved time. One student also mentioned ERL being a good option for his/her health conditions.

“I stay far from the college. Now I am saving two hours of travel”. (IN2: 120120)

##### Overall remote learning experience

The ratings for overall learning experiences clearly indicate that the students were not happy with ERL. From 138 of survey participants 61% (*n* = 84) reported their experience with emergency remote learning was terrible to bad with the mean of all ratings 3.30 (1 = love it and 5 = terrible) and standard deviation 1.19 (Online Appendix B4 and B6). But Indian students (*Mdn* = 4.0) reported higher negative ratings than Finnish students (*Mdn* = 2.50). A Mann–Whitney U-test indicates that this difference was significant *U* (Indian students = 98, Finnish students = 40) = 1326.5, *z* = -3.24, *p* < 0.01 indicating that the Indian students had worse learning experience than Finnish students when they attended emergency remote learning. In the further investigation, the study observed a relationship between the ratings given to overall remote learning experience and the challenges faced by students. A significant moderate positive correlation between these two variables (*rs* = 0.378, *p* < 0.001) indicates that students’ overall remote learning experience was associated with challenges they faced while attending ERL which means that students’ who faced more challenges had bad remote learning experiences.

##### Learning quality

The survey participants were also asked about the quality of learning they are experiencing in ERL (Online Appendix B5). Out of 137 responses, most of the students (*M* = 3.42%, SD = 1.05) rated learning quality moderately satisfactory (Online Appendix B5 and B6). There was a significant difference between the ratings given by Finnish students (*M*d*n* = 4) and Indian students (*M*d*n* = 3) for the experience of learning quality. A Mann–Whitney U-test indicates significant difference *U* (Indian students = 98, Finnish students = 39) = 1470.50, *z* = -2.21, *p* < 0.05 which indicates that Finnish students were more satisfied with the learning quality in ERL than Indian students as Indian students faced more challenges (see Table 3). Moreover, the investigation of the relationship between learning quality and the challenges (*n* = 131) revealed that they have relatively moderate negative correlation (*rs* = -0.443, *p* < 0.001), indicating that higher number of challenges were related with lower satisfaction of learning quality experienced by students.

##### Remote learning preference post-COVID

Alternatively, students were asked whether they would like to continue remote learning or want an in-person learning experience in the future post-COVID situation (1 = Full time online and 5 = No online, only in-person). Most of the students (*M* = 3.28) chose in-person learning than remote (Online Appendix B6). Indian students (*Mdn* = 4.0) were more willing to attend in-person education post-COVID than Finnish students (*Mdn* = 3.0) who showed willingness to attend remote education post-COVID. A Mann–Whitney U-test indicates that this difference was significant *U* (Indian students = 98, Finnish students = 40) = 1482.5, *z* = -2.3, *p* < 0.05 indicating that the Indian students were more inclined towards joining in-person education after lockdown than Finnish students. This can be related to higher number of challenges they faced (see Table 3). The statistical analysis of relation between challenges and the willingness towards in-person or remote learning post-COVID variables shows a significant moderate positive correlation (*rs* = 0.378, *p* < 0.001) indicating that choosing remote education, after pandemic, was associated with the number of challenges students faced during ERL which means the students who faced a greater number of challenges are likely to choose in-person education than remote.

##### Needs and wants

Participants shared various expectations for an ideal Emergency Remote Learning experience using open ended questions asked in surveys and diary study (Online Appendix A and C). Two themes that emerged from the analysis are: first, the participants wanted better interactions (*n* = 23/138 in survey and *n* = 5/7 in diary study) and secondly, they wanted futuristic technologies like holograms, virtual reality, mixed reality (*n* = 22 out of 138) that should be incorporated in ERL. Some other repeated suggestions indicated that the participants want more structured, blended (mix of face-to-face and remote), demonstrative, and shorter lectures. Participants also suggested that they would like to attend lectures in smaller groups and would be open to synchronous or asynchronous formats of classes based on the subjects.

“In physics, I usually like to see a small demonstration of the studied principles. It usually makes the lesson feel more alive. But in the online circumstances, it is understandable that the teacher does not have the required material to do so”. (D7: 011921)

“Virtual classroom, with fully immersive experience, very much like normal classrooms but from the comfort of the home”. (S138:111620)

#### Long-term effects of ERL

No significant differences between the first survey (May–June 2020: conducted after 2 months of pandemic lockdown) and the second survey (October–November 2020: conducted after 7 months of pandemic lockdown) were found in the data analysis except difference in learning quality.

The challenges reported by students from first survey (*Mdn* = 4.0) and the second survey (*Mdn* = 4.0) has no difference, a Mann–Whitney U-test *U* (First survey = 58, Second survey = 74) = 2074.5, *z* = -0.33, *p* > 0.01 clearly indicates the same. Similarly, a Mann–Whitney U-test clearly indicates that the overall remote learning experiences of students during first survey (*Mdn* = 4.0) and the second survey (*Mdn* = 4.0) has no significant difference, *U* (First survey = 62, Second survey = 76) = 1944.5, *z* = − 1.91, *p* > 0.05. Even the benefits of ERL stated by the participants in the first survey (*M* = 2.92, *SD* = 0.94) and the second survey (*M* = 3.31, *SD* = 0.85) did not indicate any significant difference in a t-test, *t* (144.67) = − 2.51, *p* >  0.05). Only the learning quality of ERL reported by students from first survey (*Mdn* = 4.0) and the second survey (*Mdn* = 3.0) has significant difference, A Mann–Whitney U-test *U* (First survey = 61, Second survey = 76) = 1694.5, *z* = − 2.84, *p* < 0.01 indicates that the quality of learning decreased over time.

On the contrary, qualitative insights reveal change of approaches towards ERL. The participants mentioned that, at first, they were uncomfortable with the ERL and reported disengagement at the beginning of the pandemic (71.43%, *n* = 5 out of 7). But as the days passed, they got adjusted with the new way of learning and the way to manage their schedules. Some participants also mentioned the techniques they used for accepting ERL. For example, rearranging the room, self-motivation, etc. Another long-term effect noted by the participants (71.43%, *n* = 5) was a ‘carefree attitude’ towards ERL. They observed that the attendance in a class has decreased drastically by 50% and the reasons stated as self-procrastination, boredom, and longer duration of lectures.

“First it was difficult then regularised”. (IN2: 120120).

“Initially tried to read as much as I could. I started getting anxious as I wasn't performing. Mid September regained the focus”. (IN5: 102420).

“Earlier when we started there were 120 students in a class. Now it’s reducing day by day. 70 or max 80 out of 120 attend the class. Some students are least bothered”. (IN2:120120).

“Time of my classes changed and teacher kept on extending it. I could not take it more last week i made a point and not attend that class”. (D5: 102420)

#### Impacts on engagement

Apart from understanding the challenges and various experiences related to ERL, this study also probed student engagement in ERL (see Fig. 2) in the diary study. Self-reported quantitative and qualitative data on engagement factors: skills, emotion, participation, and performance (Dixson, 2015) were collected separately during 5 days of diary study and then analysed cumulatively to recognize overall student engagement in ERL.

##### Skills

Throughout the 5 days of diary study and attending on an average of around three lectures per day (*M* = 2.85, see Table 2), all the seven participants (77%, *n* = 40 out of 52 entries) showed a positive approach towards improving their skills and performance during the emergency remote learning. Indian students (82%, *n* = 18 out of 22 entries) showed more inclination towards putting efforts to improve their skills and knowledge than Finnish students (73%, *n* = 22 out of 33 entries). In general, all participants noted efforts to prepare notes, doing preparation for presentations or assignments, familiarizing themselves with the course subject beforehand, reading pre-assigned articles, and practicing in group presentations.

“Today I prepared notes for 3 lectures, also discussed somethings out of topic regards to extra curricular, was attentive for mostly all lectures”. (D2: 130320)

“Done pre demonstration testing and debugging”. (D1: 120720)

“Yes, I read the non-obligatory articles and familiarized myself with the lecture topic before the class”. (D6: 011221)

##### Emotion

As emotion plays an important role in the learning engagement (Rajabalee & Santally, 2020), the study investigated the emotional states of the participants by analysing their positive or negative approach towards learning, their willingness to put efforts, applying knowledge to life and their desire to learn (Dixson, 2015). All the participants showed these emotions in their diary entries (74%, *n* = 40 out of 54 entries) by reporting excitement/positiveness if they learned something interesting or by indicating negativeness towards the class in their diary entry. Reasons behind negative responses (22.22%, *n* = 12 out of 54 entries) were uninteresting presentation, non-interactive way of teaching, or disappointing lecture delivery etc. Indian students (76%, *n* = 19 out of 25 entries) were slightly more positive and emotionally active than Finnish students (72%, *n* = 21 out of 29 entries).

“I had a lot of fun today. The conference was a change and moved away from the monotonous lecture sessions. I was introduced to new terms and studies and it was quite engaging”. (D5: 110520).

“I was excited about this course regardless of the learning environment since it was a practical course. There were many aspects of the course/skills that I can apply and improve for my future professional life”. (D6: 011521)

##### Participation

Among other factors of the engagement, the result of the ‘participation’ is the lowest. Only 45% (*n* = 23 out of 51) of total the entries indicated that the students were participating actively during ERL. There was no significant difference between Indian (43%, *n* = 10 out of 23 entries) and Finnish (46%, *n* = 13 out of 28 entries) students’ effective contribution in the lectures they attended during the diary study. The reasons behind lower participation were stated as, detached and non-interactive form of the lectures, technical difficulties, or no concentration, etc.

“No participation at all and I wasn't paying attention half the time. Every time I tried to follow the lecture 10 min after I was drifting away. It was very difficult to focus”. (D7: 011921)

“I haven't participated actively as it was a lecture-based session, I listened and followed the presentation”. (D6: 011321)

##### Performance

All participants reported good ratings for their accomplishment in all courses. The cumulative data analysis shows that 5 out of 7 (71%) participants gave more than 3 ratings (*M* = 3.49, *SD* = 0.76) to their performance in the classes they attended during 5 days of diary study and Indian students (*M* = 4.13, *SD* = 0.5) assessed their performance higher than Finnish students (*M* = 3.00, *SD* = 0.49) on the scale of 1 to 5.

##### Summarizing engagement

The cumulative analysis of skills (77% favourable), emotion (74% positive), participation (45% of entries) and performance (71% higher ratings) can state that the participants were moderately engaged. The country specific data shows that Indian students put more effort into learning skills (Indian 82% and Finnish 72%), were emotionally positive (Indian 74% and Finnish 71%) and rated their performance slightly higher (Indian *M* = 4.13 and Finnish *M* = 3) than Finnish students. On the contrary, though participation during attending ERL courses was low, Finnish students’ contribution was little more (46%) than Indian students (43%).

## Discussion

When the universities around the world decided to go full online to reduce the spread of COVID-19, they simply moved physical teaching materials to online with the aim to teach the curriculum (Aristovnik et al., 2020), resulting in an unique pedagogical shift (Aristovnik et al., 2020; Milman, 2020; Ranta et al., 2020; Rashid & Yadav, 2020) called Emergency Remote Learning (Rahiem, 2020; Schultz, 2020; Vollbrecht, 2020). It affected students’ life to the core. The regular interactions and communications from joining the lectures to attending the exams were shifted online (Toquero, 2020). This two-fold study found that the characteristics of the ERL, a make-shift non-standardized solution, non-structured learning experience, etc. affected its acceptance among students. The study also reports varied insights like challenges and other experieces along with long term effects, and learning engagement shared by students from Finland and India from their first-hand experience of using ERL for higher education.

### Characteristics of ERL

ERL is definitely a one-of-a-kind method which has differences and similarities with its pre-COVID versions (Aristovnik et al., 2020; Schultz, 2020), like distance learning or E-learning etc. (see Table 1). The first characteristic of the ERL, as the name suggests, is that it is an improvised remote learning method (Rahiem, 2020). The investigation of semi-structured interviews in this study indicated that ERL was a compulsory and sudden provision offered to the students. This hurried transaction impacted students' acceptance and sparked a concern towards ERL (Mishra et al., 2020; Rahiem, 2020; Ranta et al., 2020; Riley & McNeil, 2020).

The second characteristic of the ERL is the use of third-party software for interaction. Instead of using conventional MOOC channels the students were asked to use third party tool/s like Zoom, Microsoft Teams, Discord, WhatsApp, or/and Google Meets etc. (Rahiem, 2020). These software were not necessarily equipped with the tools required to create a pleasant, interactive, safe, and engaging learning experience for the higher education curriculum. The students reported that they were expected to use combination of two to four software (M = 3.14, SD = 0.69) to fulfil different needs of remote learning (Rahiem, 2020) like- video lecturing, answering doubts, and interacting with peers etc. Hence, limitations of third-party software and related technical issues were stated as the highest reasons behind lower concentration in ERL which also reflected in the poor ratings given by the participants to the overall learning experience of ERL.

Additionally, no structure was followed while conducting courses in ERL, resulting in low-motivation, unsatisfactory experience, and pessimistic emotions (Kapasia et al., 2020) in students. The students mentioned that each course was designed and conducted distinctively as per individual teacher’s choice and their readiness towards ERL. Instead of interactive and well-designed online courses, the students experienced monotonous or non-interactive (Schultz, 2020) lectures. The students also reported teachers’ inclination towards self-study rather than conducting synchronous or asynchronous online lectures (Hodges et al., 2020). The non-structured ERL experience resulted in creating carefree attitude in students and lesser satisfaction towards learning quality which was seen as a long-term effect of ERL.

Another distinct characteristic of ERL is no-standardisation of incorporating curriculum into a remote learning environment. Usually the content for e-learning or online learning platforms is developed and designed over the course of 4 to 6 months (Morris, 2020; Pouezevara & Horn, 2016). But a distinct and unique trend was observed in the ways of incorporating regular curriculum into the ERL environment. Some courses followed the exact same curriculum for the semester, resulting in longer course durations which was one of the biggest reasons behind increased screen time, fatigue, disappointments, and bad learning experiences among students. Whereas other courses cut down the curriculum drastically causing lower satisfaction of learning quality.

Generally, MOOC or distance learning courses are conducted in shorter duration or in parts (Hodges et al., 2020; Mishra et al., 2020) and the students usually spend around 3 to 4 hours weekly on actual lectures with flexibility in course completion time. As course duration is an important factor of remote learning experience which in contemporary MOOC courses influences course enrolments and course completions, the longer duration of course has evidently resulted in dropouts and lesser course enrolments in previous studies (Jordan, 2015). Surprisingly, the current study found that the implementation of the full curriculum in ERL forced prolonged durations (Vollbrecht, 2020) and the students spent around 6 to 10 hours per week attending classes resulted in disappointment towards overall learning experience and boredom.

### Students’ experiences, engagement, challenges, and needs

The students faced various challenges, such as distractions in home-learning setup, problems in communicating with peers and teachers, and space issues while attending university courses using ERL. But the highly mentioned challenges were low concentration, difficulties in managing schedule, bad internet, and pessimistic emotion (Mishra et al., 2020) and there were many reasons behind these challenges which were found in the qualitative insights. For example, (1) sudden shift to online content resulted in difficulty in managing schedules, (2) technological limitations of third-party tools caused monotonous lecture delivery and problems in communicating with peers, (3) non-structured ERL caused boredom, low-motivation and pessimistic emotions, and (4) distractions and space issues in home-setup resulted in low-concentration issues in the students.

The challenges which students faced while attending ERL affected their experiences such as satisfaction towards learning quality, overall remote learning experience and even choosing remote education as an option for future. These impediments are crucial for future of remote learning as they can result in frustrations, reduced grades, negative approach (Aristovnik et al., 2020, Baloran, 2020), and can create a hurdle for accepting technological advancement in education domain.

Moreover, the study revealed that the students were moderately engaged in ERL. As engagement is a key to measure course quality and effectiveness (Rajabalee & Santally, 2020; Robinson & Hullinger, 2008), the study investigated students’ skills, performance, emotions, and participation (Dixson, 2015) to investigate their engagement in ERL. The study found that the students were active, tried to improve their skills, performed moderately but did not participate much in the ERL classes. As students’ low-participation can be associated with challenges like technical constraints, non-interactivity, and low-concentration, the study can state that the students’ engagement was affected by the challenges they faced.

Students also stated their needs and proposed interesting ideas to improve ERL experiences. The most common suggestion was providing better tools and software for improved interactions, as limitations of ERL tools was the main reason behind most of the challenges. Some students suggested incorporating new technological advancement (Sahi et al., 2020), like virtual reality in remote learning where interactions can be natural and easy. Students indicated the need for more structured curriculum, shorter lectures, and organised learning experience. They also suggested making a smart use of synchronous and asynchronous learning where theoretical subjects can be taught using asynchronous methods whereas practical and skill-based subjects can be learned using active synchronous methods.

Conclusively, challenges, unsatisfactory experiences, moderate engagement, and limitations of ERL had negative effects on students’ learning experiences which subsequently formed disappointment towards ERL.

### Short-term and long-term effects

Analysis of longitudinal study evidently depicted that there was no significant difference between students’ short-term (at the beginning of pandemic lockdown) and long-term (after 10 months of pandemic lockdown) experiences with ERL except reduced satisfaction towards learning quality. Longer duration of courses, unstructured course and challenges while attending ERL resulted in decreasing satisfaction towards learning quality. On the other hand, the students showed low interest in accepting ERL at the beginning of the pandemic, but they got adjusted and figured out their own way of adjusting with the new normal. But eventually, the low satisfaction of learning quality reflected in decreased class attendance as they were bored and less motivated.

### Similarities and differences of Indian and Finnish students

Although they come from different geographics, development status and technical advanced locations, students from both the countries reported a higher number of challenges and disappointments towards ERL. They equally found it difficult to focus, hard to manage schedules, and felt distracted. Indian students who attended synchronous lectures, faced internet issues, and found it hard to communicate with their peers. They were also unsatisfied with the longer duration of the lectures resulting in boredom towards ERL.

Quantitative analysis represented that the Indian students faced more challenges like internet issues, communication with peers, keeping schedule and they were distracted while attending ERL since they attended longer and synchronous courses (see Table 2). The higher number of challenges affected in their experiences with ERL which resulted in disappointment about overall learning experience, less satisfaction towards learning quality and inclination towards refusing remote education post-COVID.

On the other hand, Finnish students felt more isolated and reported technical and teaching related challenges as they attended asynchronous or self-study-oriented courses and the structure of the courses varied as per teachers’ teaching styles. It is also possible that as most of the Finnish participants came from technical disciplines like electrical engineering or environmental engineering compared to Indian students who came from sociology or management disciplines (see Table 2), their requirements of learning technological and practical subjects were not fulfilled in the non-structured rudimentary ERL setup eventually resulted in unsatisfactory experiences.

Though students from both the countries did not find any prominent benefits of ERL, ‘no travel time’ and attending classes from the ‘comfort of home’ were some of the frequent mentions in qualitative insights. But as these benefits are more related to ‘location’ factor which can be applicable to any other remote method (see Table 1), they do not add any significant insights towards ERL.

However, insights on the ‘engagement’ factor revealed that Indian students put in more effort, performed better, and had more positive emotions. However, both Indian and Finnish students equally showed less interest in the participation which can be tied to the technically challenging (Mishra et al., 2020; Rashid & Yadav, 2020) and non-interactive characteristics of ERL. Based on these facts it can be stated that both were moderately engaged with ERL.

### What student wants: guidelines

Some unique findings and distinctive patterns were found during this pragmatic study conducted for the 10 months of pandemic lockdown and as the world is still under the shadow of COVID-19 crisis, a lot of these findings not only seem relevant but can be useful as guidelines for present practices and the coming future. The guidelines recommended in this section can be seen as improvements for emergency remote learning methods based on the challenges, experiences, and engagement insights shared by the students in this study. Educators, technology experts, and educational institutes can use these guidelines to develop a better version of remote learning experience.

- Educational institutes are advised to run internal investigations on ERLs to take steps towards contextual enhancements and structured experiences for all courses.
- The retrospective of current ERL model will help developing a more standardized version for future use. The standardization can be in the form of course material, assessment options, duration of a lecture, and interactivity, etc. These guidelines will not only create uniformity and equal education quality (Rashid & Yadav, 2020) but can also improve the effectiveness of ERL and eventually help to overcome challenges reported by students
- To improve the engagement, with the help of instructional designer (Morris, 2020; Riley & McNeil, 2020; Schultz, 2020; Vollbrecht, 2020), the educational institutes should create a repository of well-planned and structured courses which can be used for future ERL. This will also help the institutions to adopt a hybrid model for post-COVID era to accommodate students from any part of the world.
- Standardizing the duration (Mishra et al., 2020) of each lecture and making them shorter will reduce the daily screen time and will help students to manage their schedules.
- ERL should be designed as a blend of synchronous and asynchronous learning experience (Schultz, 2020; Vollbrecht, 2020). A live session on synchronous video lecture can be a better option to teach technical or skill-based subjects. Whereas asynchronous learning can be implied for theory-based subjects with self-study options. The guidelines based on subjects and curriculum should be created for teachers.
- Instead of conducting courses on third party software like Zoom, Microsoft Teams, or Google Meet etc. (see Section 7.1), the higher educational institutes can utilise their own MOOC platforms or can collaborate with commercial MOOC channels to offer a secured and familiar comprehensive solution for ERL. Since most MOOCs comprise mini tools such as- built in chat, video recording and saving facility, assessment options like quizzes, polls, and assignment submission under one roof, ERL conducted using MOOC channels can also help improving  communication, interactivity, and positiveness.
- Institutions should incorporate technological innovations for future ready versions of ERL (Riley & McNeil, 2020; Sahi et al., 2020; Virtanen et al., 2017). For example, using immersive technology like virtual reality (VR) for less disruptive and highly interactive experience, video chat rooms, open platforms for life-like social experience, and webcasting etc. can be some of the ideas.

Finally, the educators and higher education institutes need to think beyond traditional ways of teaching and should avoid the rigidness in converting conventional teaching methods into ERL. It is time to make yet another pedagogical shift by updating teaching and being adaptive towards current and future technology advancements.

## Conclusion

The COVID-19 pandemic and resulting lockdown (UNESCO, 2020) has drastically changed the lives of students enrolled in higher education from Finland and India (Gloster, 2020; Mishra et al., 2020). One of the primary shifts was experienced in the form of Emergency Remote Learning (Milman, 2020; Schultz, 2020) and this temporary make-shift solution has proved to be the most challenging part of students’ lives (Riley & McNeil, 2020; Vollbrecht, 2020). During the uncertain pandemic, it is still unknown how long the use of ERL will be continued or what kind of problems we may face in the future, so educational institutes and students must prepare for such contingency. In this regard, the present study provides a methodical 2-part approach. The first part compares conventional remote, distance, virtual, and e-learning etc. methods with ERL and the second part investigates relevant insights of challenges and experiences faced by students while adopting ERL. The study also provides insights on students’ learning engagements. The investigation was conducted using mix methods of surveys, semi-structured interviews, and diary study with participants from both India and Finland.

The comparison between ERL and other conventional online learning methods highlighted few similarities and differences. As most of the conventional methods are designed and developed with efforts, mostly representing shorter subject-oriented courses, ERL stands out to be a make-shift provision with non-standardised and non-structured method of remote learning (Toquero, 2020; Vollbrecht, 2020). The instant shift to ERL made students disappointed and emotionally pessimistic (Edelhauser E., 2020). The non-standardisation of ERL caused distinctive experiences like longer duration of courses and monotonous lecture delivery which caused frustrations and low motivation in students. Similarly, the use of third-party tools in ERL created technological limitations resulting in isolation, communication issues, and technical difficulties among students.

The students reported various challenges they faced while attending courses using ERL. Students found it hard to manage their schedules though they saved a lot of time on traveling. They also felt that they got distracted very often resulting in lower concentration and boredom towards ERL. They faced internet issues and found it hard to communicate with their peers. These challenges not only resulted in an average satisfaction towards quality of education and a disappointment towards overall learning experience with ERL, but it also had a strong negative effect on students’ willingness to continue remote learning post-pandemic.

On the contrary, students did not mention any prominent benefits of ERL. Correspondingly, no significant long-term effect of ERL is discovered except decreased satisfaction with learning quality. But the students’ attitude towards ERL show two diverse paradigms in longitudinal study. First, the students started accepting and adjusting with ERL in the longer run, but secondly, they also felt dejected and careless resulting in decreased attendance in the classes.

Geographic factors played an important role in the adoption of ERL. The study found that Indian students faced higher number of challenges and only Indian students faced higher network issues. They also attended more back-to-back synchronous classes for longer hours. This resulted in boredom, lower concentration, and distractions. These challenges affected their experiences with ERL resulting in disappointment towards overall learning experience, unsatisfaction of learning quality, and even decreased their willingness to choose remote education in the future. On the other hand, Finnish students felt more isolated as they were more concerned about the technical difficulties and the non-structured, asynchronous methods of teaching which affected their emotions and concentration. Moreover, students showed moderate engagement and low participation due to various challenges they faced.

The study discovered that the students want improved interactivity, better tools, and adaption of futuristic technology in remote learning. The findings related to challenges, experiences, and learning engagement indicated by students resulted in guidelines for educators and educational institutes. Standardization to improve interactivity, communication, structured content, shorter and uniform lectures (Riley & McNeil, 2020; Vollbrecht, 2020), blended synchronous and asynchronous learning experience, use of secured and familiar platforms like MOOC, and adapting new technology (Sahi et al., 2020) are some of the guidelines that can improve learning experiences, reform educational standards, and establish better remote education in future.

The study fills the gap between understanding the characteristics of ERL in comparison with conventional remote learning methods like online learning, distance learning, and virtual learning and its effects on students' learning experiences. The key importance of the study lies in its structure and method, as it compares longitudinal quantitative and qualitative first-hand experiences of students from various disciplines and totally different geographical backgrounds which is unique in itself. The study shown that the challenges faced by students from diferrent backgrounds while attending ERL, can affect their experiences and overall attitude towards accepting remote education in future.

Furthermore, since there are very few studies done on Finnish university level students during the pandemic, the contribution of this study becomes valuable for further research. The study intends to guide educators and higher education institutes towards the future direction of education and technology advancements making them future ready for similar situations for example, calamity prone countries/regions like Hong Kong (cyclone prone), Japan (earthquakes), and Uniteed States of America (hurricanes). An improved form of Emergency Remote Learning can be also useful in the countries where women are still restricted to go to colleges.

## Limitations and further study

Due to limitations on mobility and communications during the pandemic, reaching out to the participants were restricted resulting in only 138 participants from both the countries in the survey. A higher number of data samples might reveal different patterns and findings. The similar limitation also occurred during diary study. It will be useful to plan a more extensive study with more participants focusing on the longitudinal data. The data on learning quality and interactivity of ERL was not analysed separately for discipline-specific subjects (theoretical and practical or skill-based subjects). Maybe a qualitative investigation can throw light on challenges and experiences of learning practical or demonstrative subjects in ERL. The study only focused on the students’ perspective, but since teachers play an important role in student’s learning engagement (Marx et al., 2016), further study will be required to capture the needs, challenges, and experiences of the university level teachers.

## Supplementary Information

Below is the link to the electronic supplementary material.

Supplementary file 1 (DOCX 74 kb)

## Data Availability

Stored in safe environment available upon request.

## Footnotes

1 Mentioned universities and colleges do not represent participants’ affiliation and are done purposely for data protection reasons.

2 As stated by Indian govenment's webpage: https://digitalindia.gov.in/writereaddata/files/6.Data%20Protection%20in%20India.pdf

## References

[CR1] Ali A, Ramay M, Shahzad M (2011). Key factors for determining student satisfaction in distance learning courses: A study of Allama Iqbal Open University (AIOU) Islamabad, Pakistan. The Turkish Online Journal of Distance Education.

[CR2] Aristovnik, A., Keržič, D., Ravšelj, D., Tomaževič, N., & Umek, L. (2020). Impacts of the COVID-19. In *Pandemic on life of higher education students: A global perspective.*10.20944/preprints202008.0246.v2. 10.1016/j.dib.2021.107659 PMC8634691 34869802

[CR3] Bailey JE, Pearson SW (1983). Development of a tool for measuring and analyzing computer user satisfaction. Management Science.

[CR4] Baloran ET (2020). Knowledge, Attitudes, Anxiety, and Coping Strategies of Students during COVID-19 Pandemic. Journal of Loss and Trauma.

[CR5] Beek, M. (2011). Introduction: What is virtual learning? A Mackinac Center Report.

[CR6] Berg, D. (2016). Distance learning. In *Encyclopaedia Britannica*. Retrieved December 21, 2020, from https://www.britannica.com/topic/distance-learning/additional-info.

[CR7] Cao W, Fang Z, Hou G, Han M, Xu X, Dong J, Zheng J (2020). The psychological impact of the COVID-19 epidemic on college students in China. Psychiatry Research.

[CR8] Cohen, L., Manion, L., & Morrison, K. (2017). Research methods in education (pp. 265-266). ProQuest Ebook Central https://www.ebookcentral.proquest.com

[CR9] Dam, R. F., & Siang, T. Y. (2020). Affinity diagrams—learn how to cluster and bundle ideas and facts, Interaction design foundation. Retrieved November 30, 2020, from https://www.interaction-design.org/literature/article/affinity-diagrams-learn-how-to-cluster-and-bundle-ideas-and-facts.

[CR10] Digital Initiative. (2021). Harvard Business School. Retrieved January 2, 2021, from https://digital.hbs.edu/.

[CR11] Dixson M (2015). Creating effective student engagement in online courses: What do students find engaging?. Journal of the Scholarship of Teaching and Learning.

[CR12] Dziuban C, Moskal P, Thompson J, Kramer L, DeCantis G, Hermsdorfer A (2015). Student satisfaction with online learning: Is it a psychological contract?. Online Learning.

[CR13] Edelhauser E, Lupu-Dima L (2020). Is Romania prepared for eLearning during the COVID-19 Pandemic?. Sustainability..

[CR14] Ethics Committee Tampere. (2010). Responsible conduct of Research. Tampere Universities. Retrieved January 14, 2021, from https://www.tuni.fi/en/research/responsible-research.

[CR15] EU-GDPR. (2016). EU General Data protection Regulations. Privacy-regulation-EU. Retrieved May 30, 2020, from https://www.privacy-regulation.eu/en/index.htm.

[CR16] Finnish Government. (2020). Government Communications Department Ministry of Education and Culture Ministry of Social Affairs and Health, Government, in cooperation with the President of the Republic, declares a state of emergency in Finland over coronavirus outbreak. Finnish Government. Retrieved November 28, 2020, from https://valtioneuvosto.fi/en/-/10616/hallitus-totesi-suomen-olevan-poikkeusoloissa-koronavirustilanteen-vuoksi.

[CR17] Gloster L (2020). Impact of COVID-19 pandemic on mental health: An international study. PLoS ONE.

[CR18] Handelsman M, Briggs W, Sullivan N, Towler A (2005). A measure of college student course engagement. The Journal of Educational Research..

[CR19] Hodges, C., Moore, S., Lockee, B., Trust, T., & Bond, A. (2020). The difference between emergency remote teaching and online learning. *Educause Review*. Retrieved December 20, 2020, from https://er.educause.edu/articles/2020/3/the-difference-between-emergency-remote-teaching-and-online-learning#fn3.

[CR20] IGNU. (2021). Gyan Darshan, Indira Gandhi National Open University, India. Retrieved January 7, 2021, from http://ignou.ac.in/ignou/aboutignou/broadcast/2.

[CR21] IIT Bombay. (2020). *Decision of senate with regard to closure of semesters and academic year, 2019–2020*. Indian Institute of Technology Bombay. Retrieved January 7, 2021, from https://www.iitb.ac.in/en/decision-senate-regard-to-closure-semesters-and-academic-year-2019-20.

[CR22] Jordan K (2015). Massive open online course completion rates revisited: Assessment, length and attrition. The International Review of Research in Open and Distributed Learning.

[CR23] Jung H-J (2014). Ubiquitous learning: Determinants Impacting learners’ satisfaction and performance with smartphones. Language Learning & Technology.

[CR24] Kapasia N, Paul P, Roy A, Saha J, Zaveri A, Mallick R, Barman B, Das P, Chouhan P (2020). Impact of lockdown on learning status of undergraduate and postgraduate students during COVID-19 pandemic in West Bengal, India. Children and Youth Services Review.

[CR25] Kuh GD (2003). What we’re learning about student engagement from NSSE: Benchmarks for effective educational practices. Change: the Magazine of Higher Learning.

[CR26] Marsh H (1982). SEEQ: A reliable, valid, and useful instrument for collecting Students’ evaluations of university teaching. British Journal of Educational Psychology..

[CR27] Marx A, Simonsen J, Kitchel T (2016). Undergraduate student course engagement and the influence of student, contextual, and teacher variables. Journal of Agricultural Education..

[CR28] Milman N. (2020). Pandemic pedagogy. *Phi Delta,* Kappan. https://kappanonline.org/pandemic-pedagogy-covid-19-online-milman/.

[CR29] Mishra L, Gupta T, Shree A (2020). Online teaching-learning in higher education during lockdown period of COVID-19 pandemic. International Journal of Educational Research Open.

[CR30] MOOC.fi. (2021). University of Helsinki, Finland. Retrieved January 11, 2021, from https://www.mooc.fi/.

[CR31] Morris, M. (2020). A pedagogy of transformation for times of crisis – OEB Insights. *OEB Global*. Accessed 14 January 2020.

[CR32] Mundy, D., & Wilcox, C. (2020). What is student engagement in lockdown? Teaching academy, University of Hull. Retrieved January 12, 2021, from https://www.hull.ac.uk/choose-hull/study-at-hull/teaching-academy/news/what-is-student-engagement-in-lockdown.

[CR33] Nowell LS, Norris JM, White DE, Moules NJ (2017). Thematic analysis: Striving to meet the trustworthiness criteria. International Journal of Qualitative Methods.

[CR34] O’Flaherty, K. (2020). Beware Zoom users: Here’s how people can Zoom-bomb your chat. Forbs. Retrieved October 23, 2020, from https://www.forbes.com/sites/kateoflahertyuk/2020/03/27/beware-zoom-users-heres-how-people-can-zoom-bomb-your-chat/?sh=63bc1e4618e2.

[CR35] Official Statistics of Finland. (OSF). Use of information and communications technology by individuals [e-publication]. ISSN=2341-8710. Statistics Finland (referred: 6.12.2020). http://www.stat.fi/til/sutivi/index_en.html.

[CR36] Pouezevara, S., & Horn, L. (2016). *MOOCs and online education: Exploring the potential for international educational development*. RTI Press Publication No. OP-0029-1603. RTI Press. 10.3768/rtipress.2016.OP.0029.1603.

[CR37] Rahiem M (2020). The emergency remote learning experience of university students in Indonesia amidst the COVID-19 crisis. International Journal of Learning, Teaching and Educational Research.

[CR38] Rajabalee YB, Santally MI (2020). Learner satisfaction, engagement and performances in an online module: Implications for institutional e-learning policy. Education and Information Technologies.

[CR39] Ramsden P (1991). A performance indicator of teaching quality in higher education: The course experience questionnaire. Studies in Higher Education..

[CR40] Ranta M, Silinskas G, Wilska T-A (2020). Young adults' personal concerns during the COVID-19 pandemic in Finland: an issue for social concern. International Journal of Sociology and Social Policy.

[CR41] Rashid S, Yadav S (2020). Impact of Covid-19 pandemic on higher education and research. Indian Journal of Human Development.

[CR42] Reznik A, Gritsenko V, Konstantinov V (2020). COVID-19 fear in eastern Europe: Validation of the fear of COVID-19 scale. International Journal of Mental Health and Addiction.

[CR43] Riley J, McNeil WC (2020). Student experiences of emergency remote teaching: Impacts of instructor practice on student learning, engagement, and well-being. Journal of Chemical Education.

[CR44] Roblyer M, Wiencke W (2004). Exploring the interaction equation: Validating a rubric to assess and encourage interaction in distance courses. Journal of Asynchronous Learning Networks.

[CR45] Robinson C, Hullinger H (2008). New benchmarks in higher education: Student engagement in online learning. Journal of Education for Business.

[CR46] Rosala, M. (2019). How to analyze qualitative data from UX research: Thematic analysis. *NN-Nielsen Norman Group*. Retrieved November 12, 2020, from https://www.nngroup.com/articles/thematic-analysis/.

[CR47] Sahi PK, Mishra D, Singh T (2020). Medical Education amid the COVID-19 pandemic. Indian Pediatrics.

[CR48] Sahu P. (2020). Closure of Universities Due to Coronavirus Disease 2019 (COVID-19): Impact on Education and Mental Health of Students and Academic Staff. Cureus, 12(4), e7541. 10.7759/cureus.7541 10.7759/cureus.7541 PMC7198094 32377489

[CR49] Schultz DeMers (2020). Transitioning from emergency remote learning to deep online learning experiences in geography education. Journal of Geography (houston).

[CR50] Second Life. (2020). Linden lab. Retrieved December 15, 2020, from https://secondlife.com/.

[CR51] Shahabadi MM, Uplane M (2015). Synchronous and asynchronous e-learning styles and academic performance of e-learners. Procedia—Social and Behavioral Sciences.

[CR52] St Xavier’s College. (2020). Note for students and parents (COVID-19 and Exams), *sxcbom*. Retrieved December 4, 2020, from https://sxcbom.com/2020/03/13/note-for-students-and-parents-covid-19-exams/.

[CR53] Stafford Global. (2020). *What’s the difference between online learning and distance learning*. Stafford Global. Retrieved January 20, 2021, from https://www.staffordglobal.org/articles-and-blogs/whats-the-difference-betwebeeen-online-and-distance-learning/*.*

[CR54] Stanford Online. (2021). Stanford University, CA. Retrieved January 14, 2021, from https://online.stanford.edu/.

[CR55] Swan K, Shea P, Fredericksen E, Pickett A, Pelz W, Maher G (2000). Building knowledge building communities: consistency, contact and communication in the virtual classroom. Journal of Educational Computing Research.

[CR56] TENK. (2012). Guidelines for ethical review in human sciences. Finnish National Board on Research Integrity. TENK. Retrieved October 11, 2020, from https://tenk.fi/en/advice-and-materials/guidelines-ethical-review-human-sciences#3_3.

[CR57] Toquero CM (2020). Emergency remote education experiment amid COVID-19 pandemic. IJERI: International Journal of Educational Research and Innovation.

[CR58] UNESCO. (2020). COVID 19 Impact of Education. Global monitoring of school closures caused by COVID-19. Retrieved January 15, 2021, from https://en.unesco.org/covid19/educationresponse.

[CR59] UN News. (2020). COVID-19: Lockdown across India, in line with WHO guidance. UN India. Retrieved January 28, 2021, from https://news.un.org/en/story/2020/03/1060132.

[CR60] University of Helsinki. (2021). Coronavirus situation at University of Helsinki. Retrieved January 27, 2021, from https://www.helsinki.fi/en/news/coronavirus-situation-at-the-university-of-helsinki.

[CR61] Virtanen MA, Kääriäinen M, Liikanen E, Haavisto E (2017). The comparison of students’ satisfaction between ubiquitous and web-based learning environments. Education and Information Technologies.

[CR62] Vollbrecht P-S (2020). Lessons learned while creating an effective emergency remote learning environment for students during the COVID-19 pandemic. Advances in Physiology Education.

[CR63] Wikipedia. (2020). List of Open Universities Wikipedia. Retrieved October 30, 2020, from https://en.wikipedia.org/wiki/List_of_open_universities.

[CR64] World Health Organization. (WHO). Coronavirus Disease (COVID-2019) Situation Reports. Retrieved December 17, 2020, from https://www.who.int/emergencies/diseases/novel-coronavirus-2019/situation-reports.

[CR65] Xiao, C., & Li, Y. (2020). Analysis on the influence of the epidemic on the education in China. In *2020 International conference on big data and informatization education (ICBDIE)*, Zhangjiajie, China (pp. 143–147).

