# Supplementary material for: What students want? Experiences, challenges, and engagement during Emergency Remote Learning amidst COVID-19 crisis
Source: Educ Inf Technol (Dordr). 2021 Oct 20;27(1):551–87. doi: 10.1007/s10639-021-10747-1 (PMC8527278; doi:10.1007/s10639-021-10747-1)
Supplement: Supplementary file 1 — (DOCX 74 kb) [file 10639_2021_10747_MOESM1_ESM.docx]

**What students want? Experiences, challenges, and engagement during Emergency Remote Learning amidst COVID-19 crisis**

***Appendix A: Survey questionnaires***

*Section 1- Personal details*

1. What is your gender? Woman, Man, Non-binary, Prefer not to say
2. Which age group you belong to? Below 18, 18 to 22, 23 to 35, 36 to 45, Above 45
3. What is your country of residence?
4. Which degree or educational program are you currently enrolled in? Bachelor's degree, Master's degree, Doctorate, Professional course, Other

*Section 2- Online Experience*

1. How has your experience been while attending courses/lectures from home? Love it, Better than physical class, Same as physical class, Not ideal, Terrible
2. How many hours on average 'per Week' did you spend attending courses/lectures online during the last month? 1 to 2 hours, 3 to 5 hours, 6 to 9 hours, 10 or more hours
3. Which courses/topics are you studying online?
4. How are the courses assessing your knowledge? (select all which are applicable)- Quiz, Offline assignments, Essays, Presentations, Discussions, Other

*Section 3- Learning Experience*

1. What are the biggest challenges you have faced when attending courses/lectures online? (select all which are relevant and/or write in 'other')
   1. Communication with classmates
   2. Communication with course supervisor
   3. Keeping a consistent, regular schedule
   4. Creating a separate space for online sessions at home
   5. General anxiety about COVID19
   6. Social isolation
   7. Presenting own work
   8. Internet connectivity
   9. Too many distractions at home
   10. Technical issues when using audio and video
   11. None- I am comfortable with the arrangement
2. Rate the following in terms of how much they benefit you while attending online courses/lectures. Used scale- Not at all, A little bit, Somewhat, Very much, Extremely
   1. No travel time
   2. More control over my schedule
   3. No Distractions during lectures/courses
   4. Overall Mental/emotional well being
   5. Easy to concentrate
   6. More time for what is important to me in my life in addition to studies (Family, friends, hobbies, exercise)
   7. Taking classes from comfort of home
3. Please mention things you like/dislike about online learning?
4. Are you satisfied with the ‘learning quality’ of online learning? 1star= Not satisfied, 5 star= Extremely satisfied
5. How interactive are your online courses/lectures on average (assuming you are using tools like Zoom, Teams etc.)?
   1. Very interactive (students ask questions, discuss the topic and solve problems as a group)
   2. Somewhat interactive (students ask questions when given a chance and can talk to lecturer after the lecture)
   3. Partially interactive (students ask questions and discuss topics using chat)
   4. Very little interactive (students reply using chat if a question is asked)
   5. Not interactive at all
   6. Other
6. Post-COVID19, after the social distancing is over and the universities/colleges/classes open again, how often would you like to attend online lectures/courses?
   1. Full time online
   2. 1-3 days per week online
   3. I want the flexibility to join virtual class whenever I need it
   4. I want to attend in-person classes for fewer hours daily and rest virtually
   5. I do not want to attend online classes at all and want them only in-person.
7. If there are no technological restrictions, how would you imagine an ideal online learning environment in the near future?

***Appendix B1***

***Appendix B2***

***
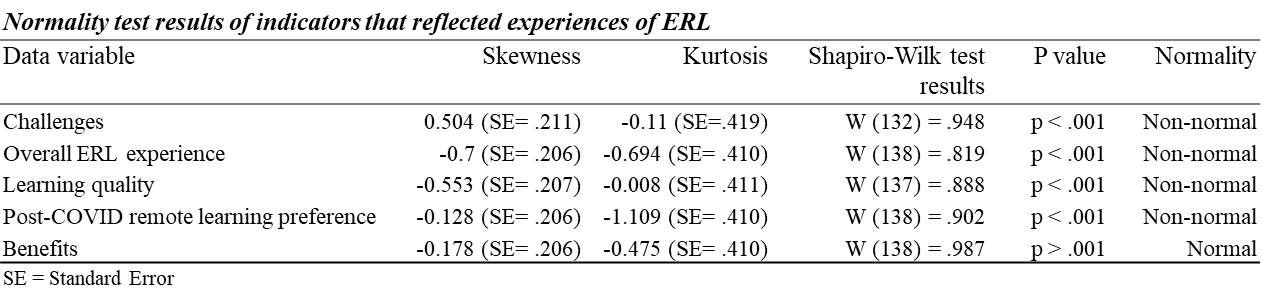
***

***Appendix B3***

***Descriptive statistics of items of benefits (n=138)***

***Appendix B4***

***Appendix B5***

***Appendix B6***

***
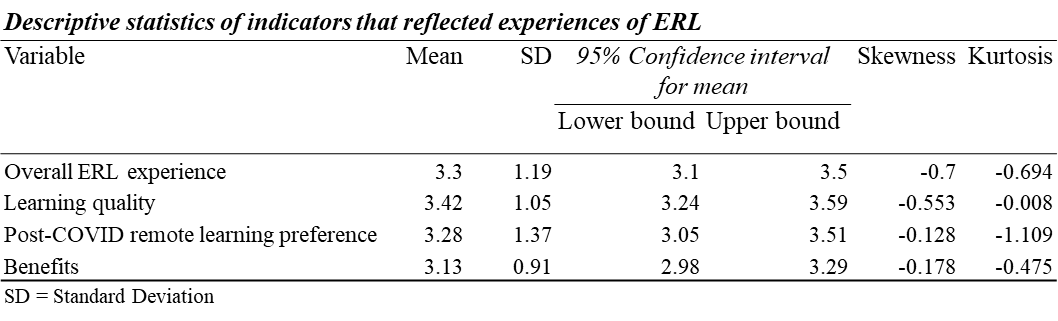
***

***Appendix C- Semi-structured interview questionnaire***

1. Current experience-
   1. Which tools do you use to attend class? (zoom, Teams etc.)
   2. Live or recorded?
   3. Is the regular curriculum taught or alternative changes are made?
   4. Which devices do you use to attend classes/courses? (PC, mobile etc)
   5. How do you manage your schedule?
   6. How many students in a class?
2. What will you say about your overall experience attending online class for the past 6 months compared to your regular experience before COVID19 lockdown?

***Appendix D- Diary study questionnaire***

Section 1- Overall experience

1. How are you feeling today? 1= Sad, Depressed, Down, 5 = Happy, Excited, Awesome, Great
2. How long did you attend classes? (combining all)
3. Rate today’s overall learning experience for all classes. 1 = lowest, 5 = highest
4. How was your overall performance in all classes (marks, assessments)? 1= Very bad, 5= Extremely well

Section 2- Subject 1^1^

1. Which class did you attend today?
2. How did you feel about online learning experience (Applying learning to life, putting efforts, want to learn etc.)
3. Did you try to improve your 'Skills/knowledge' today? (prepared notes, studied apart from lectures, being organized, was attentive etc.)
4. How was you 'Participation' (helped classmates, presented/talked about assignment, engaged in conversation, was a silent listener etc.)?
5. What were some of the challenges you faced (scheduling, concentrating, space, internet, interaction etc.)? How did you tackle them?
6. Were there any specific study needs which did not get fulfilled through online experience?
7. Rate your experience with the teacher. 1 = lowest, 5= highest
8. Mark the following statements based on today's experience in the class.

Scale- Never, Rarely, Often, All the time

- 1. My video was on
  2. I could see teacher
  3. I could see all/some of the classmates
  4. I texted/talked to other friends/relatives during lecture
  5. I did other things lunch/game/tv/checked other sites during lecture

^1^Sections 2 was repeated if the participant was attending more than 1 class per day. Name of the section then changed to 2, 3, 4…so on
